# Optimal peritumoral regions and fusion strategies for prediction of the double-expressor subtype in diffuse large B-cell lymphoma: a multi-region radiomics study

**DOI:** 10.3389/fonc.2026.1877578

**Published:** 2026-07-23

**Authors:** Qiwen Zhong, Guoxiu Lu, Jingjing Liang, Qi Peng, Ronghui Tian, Guoxu Zhang

**Affiliations:** 1 Department of Nuclear Medicine, General Hospital of Northern Theatre Command, Shenyang, China; 2 Training Base for Graduate, General Hospital of Northern Theater Command, Dalian Medical University, Shenyang, China; 3 Training Base for Graduate, General Hospital of Northern Theater Command, China Medical University, Shenyang, China; 4 School of Software, Shenyang University of Technology, Shenyang, Liaoning, China

**Keywords:** ^18^F-FDG PET/CT, deep learning radiomics, diffuse large B-cell lymphoma, double-expressor lymphoma (DEL), multi-regional analysis

## Abstract

**Objective:**

To develop a PET/CT-based radiomics model for noninvasive prediction of the double-expressor lymphoma (DEL) subtype in diffuse large B-cell lymphoma (DLBCL).

**Materials and methods:**

From January 2019 to July 2025 consecutively enrolled, 143 patients with DLBCL (55 DEL, 88 non-DEL) were randomly divided into a training set and an internal validation set in a 7:3 ratio. Radiomic features were extracted from peritumoral regions with different expansion distances (3, 5, and 10 mm) to identify the optimal peritumoral region. These features were then integrated using various fusion strategies (multi-region fusion, image fusion, and feature fusion) for combined intratumoral and peritumoral analysis. A transfer learning model built on a pretrained ResNet-50 was combined with a clinical model incorporating baseline PET features to develop three integrated models (Clinic+Rad+DL, Stacking, Ensemble). Model performance was evaluated to select the best-performing approach, and SHAP analysis was applied to enhance interpretability.

**Results:**

The Rad_MLP model achieved the best performance by integrating intratumoral and 10-mm peritumoral features, with an accuracy of 85.3%, a sensitivity of 89.5%, and a specificity of 79.2%. The positive predictive value (PPV) and negative predictive value (NPV) were 0.773 and 0.905, respectively, with an F1 score of 0.829.

**Conclusion:**

Rad_MLP, by integrating the most predictive intratumoral and peritumoral features, substantially improves the accuracy of noninvasive prediction of the DEL subtype in DLBCL.

## Introduction

Diffuse large B-cell lymphoma (DLBCL) accounts for approximately 150,000 new cases globally each year, representing about 30% of all non-Hodgkin lymphoma cases (1). Among the various subtypes of DLBCL, double-expressor lymphoma (DEL) is characterized by high expression of MYC and BCL2 proteins, accounting for 20%–30% of newly diagnosed DLBCL cases (1). Early and accurate diagnosis of DEL is crucial for patient management and prognosis. First, DEL is highly aggressive; most patients present with stage III–IV disease and exhibit higher rates of multi-organ involvement, including the bone marrow, liver, spleen, and central nervous system. Early detection may reduce the risk of systemic tumor cell dissemination and central nervous system involvement (2, 3). Second, prognosis and treatment are closely linked to DEL status: patients receiving intensified regimens such as DA-EPOCH-R (dose-adjusted etoposide, prednisone, vincristine, cyclophosphamide, doxorubicin, and rituximab) exhibit superior overall survival and progression-free survival compared with those receiving standard R-CHOP (rituximab plus cyclophosphamide, doxorubicin, vincristine, and prednisone) immunochemotherapy (4, 5). This highlights that early and precise identification of DEL is essential for achieving personalized treatment and improving patient outcomes.

¹^8^F-FDG PET/CT provides both metabolic and anatomical information; metabolic parameters such as SUVmax, MTV, and TLG reflect tumor metabolic activity (6). When integrated, these parameters enable PET/CT to achieve high sensitivity and specificity in detecting bone marrow involvement in DLBCL (93% and 98%, respectively) (7), making it an important tool for early DEL diagnosis (8). However, conventional visual interpretation of PET/CT images yields relatively lower sensitivity and specificity in the assessment of mid-treatment response (80% and 72%, respectively) (9). Radiomics, which extracts quantitative features from PET/CT images, can reveal tumor heterogeneity and improve predictive accuracy (10). Meta-analyses indicate that PET/CT-based radiomics achieves a sensitivity of 82% and a specificity of 83% (11), demonstrating excellent performance in predicting treatment response and relapse in DLBCL (12). Subsequent studies have confirmed that PET/CT-based deep learning radiomics models can effectively predict treatment response and relapse risk. Yuan et al. (13) used PET/CT images from 249 patients with DLBCL and a hybrid Conv-LSTM model to predict primary treatment failure, achieving an accuracy of 88.64% and an AUC of 0.925. Jiang et al. (14) retrospectively analyzed 684 patients and generated deep learning scores (DLS) using VGG19 and DenseNet121 architectures, yielding C-indices of 0.760/0.770 for PFS and 0.748/0.766 for OS, respectively. Cui et al. (15) used PET images from 271 patients to develop an optimized PET radiomics fusion model, achieving an AUC of 0.898 and a C-index of 0.853 for predicting 2-year time to progression. Nevertheless, current studies are limited by small sample sizes, a lack of peritumoral analysis, and the absence of DEL-specific modeling, underscoring the unmet need for the development of robust, noninvasive predictive models for DEL.

To address these gaps, this study aimed to develop a radiomics model based on intratumoral and multi-scale peritumoral PET/CT features to improve the accuracy of predicting the DEL subtype in DLBCL. Compared with previous studies, our work focused on multi-scale peritumoral regions and DEL-specific modeling. We established and validated a combined radiomics model that integrated multiple fusion strategies, incorporating the most predictive intratumoral features and optimally selected peritumoral features, ultimately enabling high-precision, noninvasive prediction of DEL.

## Materials and methods

### Study population

A retrospective analysis was conducted on 354 patients who underwent pre-treatment ^18^F-FDG PET/CT scanning at the Northern Theater General Hospital between January 2019 and July 2025 consecutively enrolled. Original PET/CT DICOM images, pathology reports, laboratory results, and treatment records were retrieved from our institution’s electronic medical record system. Staining for MYC and BCL2 was performed routinely on a validated automated platform using commercial kits; specific clone details were not retained in the archival records. DEL was defined per the 2016 WHO criteria (c-Myc ≥40% nuclear and Bcl-2 ≥50% cytoplasmic/membranous) (16), and all cases were independently scored by two hematopathologists blinded to clinical data. This study was approved by the Ethics Committee of Northern Theater General Hospital (Approval No. Y (2025) 187) and conducted in accordance with the Declaration of Helsinki. Given the retrospective nature of the study, informed consent was waived.

**Figure 1:**
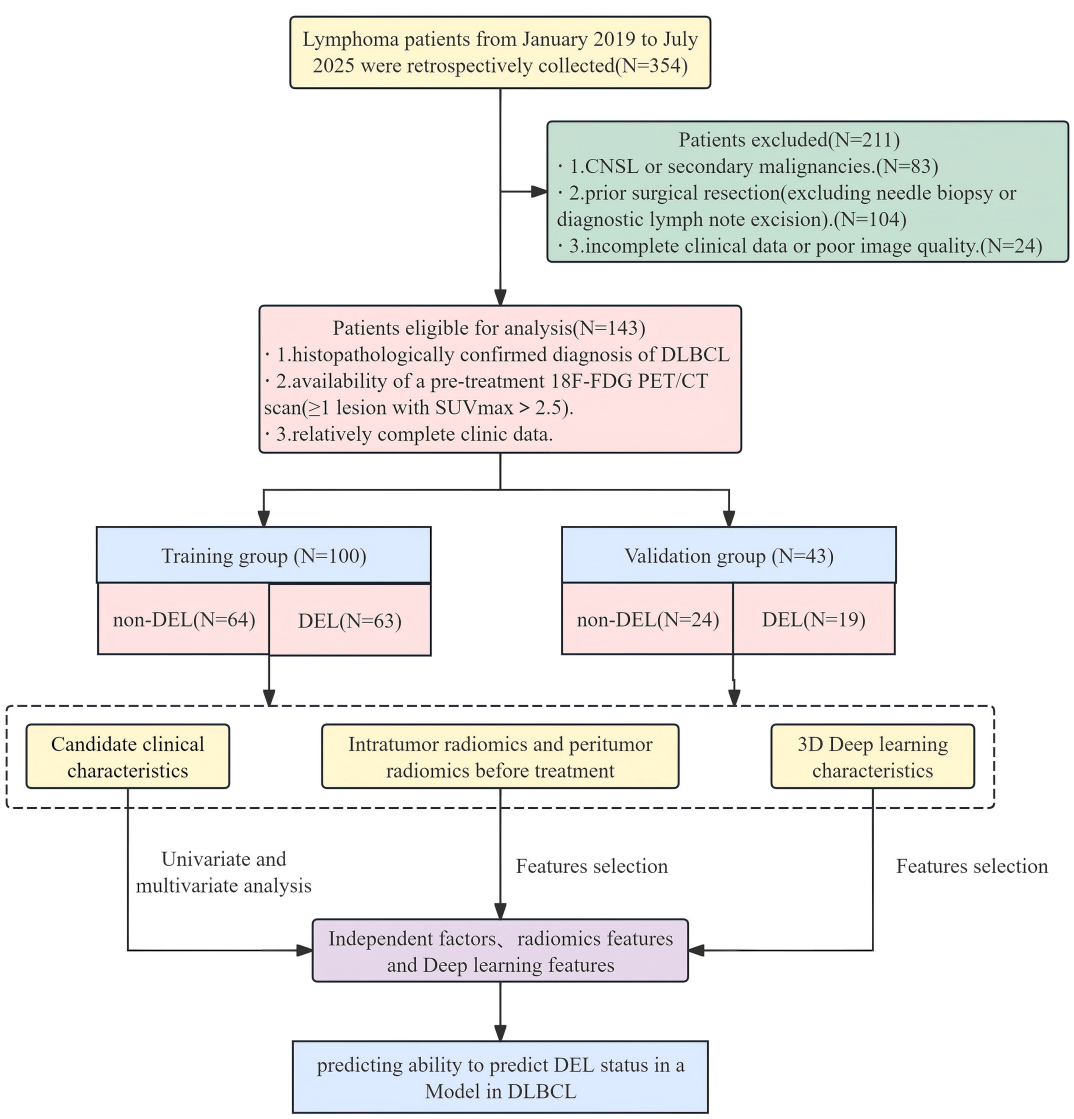
Flowchart of patient recruitment.

Inclusion criteria were: (1) pathologically confirmed DLBCL; (2) no treatment before the ^18^F-FDG PET/CT examination; (3) complete baseline PET/CT data; and (4) complete clinical information. Exclusion criteria were: (1) primary central nervous system lymphoma or concurrent malignancies (N = 83); (2) prior anti-tumor therapy (e.g., surgery, chemotherapy, radiotherapy)(N = 104); (3) poor image quality or missing clinical data(N = 24); and (4) severe cardiac, hepatic, or renal dysfunction. Ultimately, 143 patients meeting the criteria were included, comprising 88 non-DEL and 55 DEL patients. The dataset was randomly split into a training set (70%) and an internal validation set (30%) using a random seed of 42. The complete patient ID list with cohort assignment and class labels is provided in **Supplementary Table 1**. The training set was used solely for model weight fitting, while the internal validation set was used to determine the optimal peritumoral expansion size, select the best-performing classifier among candidates, and decide on the optimal fusion strategy (feature-level vs. decision-level), resulting in 100 patients (64 non-DEL, 36 DEL) in the training set and 43 patients (24 non-DEL, 19 DEL) in the validation set, as shown in **Figure 1**.

### Image acquisition and processing

All patients were scanned on one of two identical GE Discovery MI PET/CT systems and 710 in PET/CT systems(GE Healthcare, Chicago, IL, USA). PET reconstruction parameters were as follows: matrix size 256 × 256; voxel size 2.67 × 2.67 × 3.27 mm; ordered-subset expectation maximization (OSEM) with 2 iterations and 8 subsets; post-filtering with a Gaussian filter of 3.0 mm FWHM; TOF and PSF correction were applied. CT reconstruction was performed using a standard soft-tissue kernel with a slice thickness of 3.75 mm. Patients were required to fast for at least 6 hours before imaging, and blood glucose levels were confirmed to be below 11.1 mmol/L to minimize the impact of hyperglycemia on radiotracer uptake. ^18^F-FDG was produced using a GE Healthcare medical cyclotron and an automated synthesis module, and administered intravenously at a dose of 3.70–5.18 MBq/kg based on body weight. Following injection, patients rested for 50–60 minutes to allow adequate tracer uptake. Scans were performed from the base of the skull to mid-thigh, and extended to the feet if clinically indicated. The low-dose attenuation-correction CT scan acquisition parameters were as follows: tube voltage, 120 kV; tube current, 110 mA; pitch, 1.0; rotation time, 0.5 s; slice thickness, 3.27 mm (with 2-mm overlap).PET images were acquired in three-dimensional (3D) mode across 6–8 bed positions, at 1.5 min per bed position. All PET images were attenuation-corrected using CT data and reconstructed as fused PET/CT images for subsequent radiomics analysis.

### Region of interest delineation

**Figure 2:**
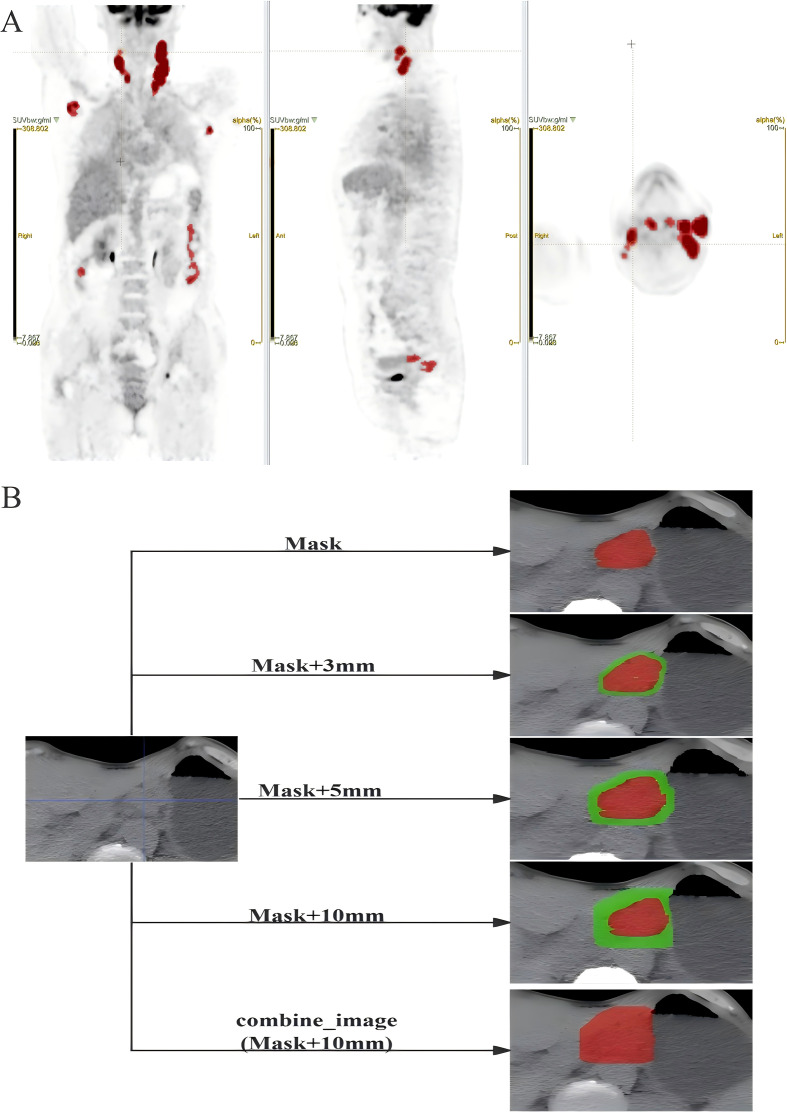
Representative PET image of a lymphoma patient and schematic illustration of peritumoral margin expansion for radiomics analysis. **(A)** Coronal, sagittal, and axial PET views (from left to right) of a representative lymphoma patient (ID: PT51360), with all target tumor lesions highlighted in magenta. **(B)** Schematic demonstration of peritumoral region construction on an axial CT slice. From top to bottom: the primary tumor mask (intratumoral region, marked in red); 3-mm peritumoral region expanded outward from the tumor boundary (green margin); 5-mm peritumoral region; 10-mm peritumoral region; and the combined region integrating the intratumoral area with the 10-mm peritumoral area (combine_image, marked in red).

All PET/CT images were resampled to a uniform voxel size of 1 × 1 × 1 mm³ and intensity-normalized to minimize bias due to resolution differences. Two experienced radiologists (with 8 and 10 years of experience in nuclear medicine imaging) manually delineated ROIs on PET/CT fusion images using LIFEx software (17) and 3D Slicer (v5.0), any discrepancies were adjudicated by a senior radiologist with more than 20 years of experience. (ICC>0.800). A total of 28 features out of 214 were retained who were independently and fully blinded to the patients’ DEL status and all clinical outcomes. For patients with multiple lesions, all detectable lesions were contoured and merged into a single patient-level union mask rather than segmenting each lesion individually or selecting only the most metabolically active dominant lesion. A representative case (patient ID: PT51360) with multifocal lesions is illustrated in **Figure 2A**.

SUVmax, MTV, and TLG were calculated for each individual lesion, and total metabolic tumor volume (TMTV) was computed as the sum of MTV across all lesions within the same patient, consistent with standard clinical practice (18, 19). Subsequently, peritumoral expansion regions were generated outward from the patient-level union mask using Python (SimpleITK, v3.6) with expansion distances of 3 mm, 5 mm, and 10 mm, yielding multi-scale peritumoral ROIs (**Figure 2B**). For quality control, all automatically generated expansion masks were reviewed slice-by-slice by two radiologists, and manual trimming was performed when necessary to exclude regions extending beyond anatomical boundaries (e.g., body wall, large vessels, bone). These expansion scales were selected based on prior multi-cancer, multi-scale radiomics studies demonstrating their effectiveness in capturing biological gradient features from the tumor margin to the surrounding microenvironment. Specifically, a 3-mm peritumoral region was shown to perform best in predicting pathologic complete response to neoadjuvant chemoradiotherapy in oral squamous cell carcinoma using MRI-based intratumoral and peritumoral radiomics (20). A 5-mm expansion performed well in a multimodal radiomics model integrating MRI, ultrasound, and mammography for differentiating between benign and malignant breast nodules (21). The 10-mm expansion was used to may potentially capture a broader tumor microenvironment (TME), including inflammation, stromal remodeling, and immune cell infiltration, and has been shown to overlap with high-risk recurrence regions in imaging studies of lymph node metastasis in tongue cancer (22). These distances were selected based on prior radiomics literature in other tumor types, and their use in DLBCL is exploratory.

### Radiomic feature extraction and model development

**Figure 3:**
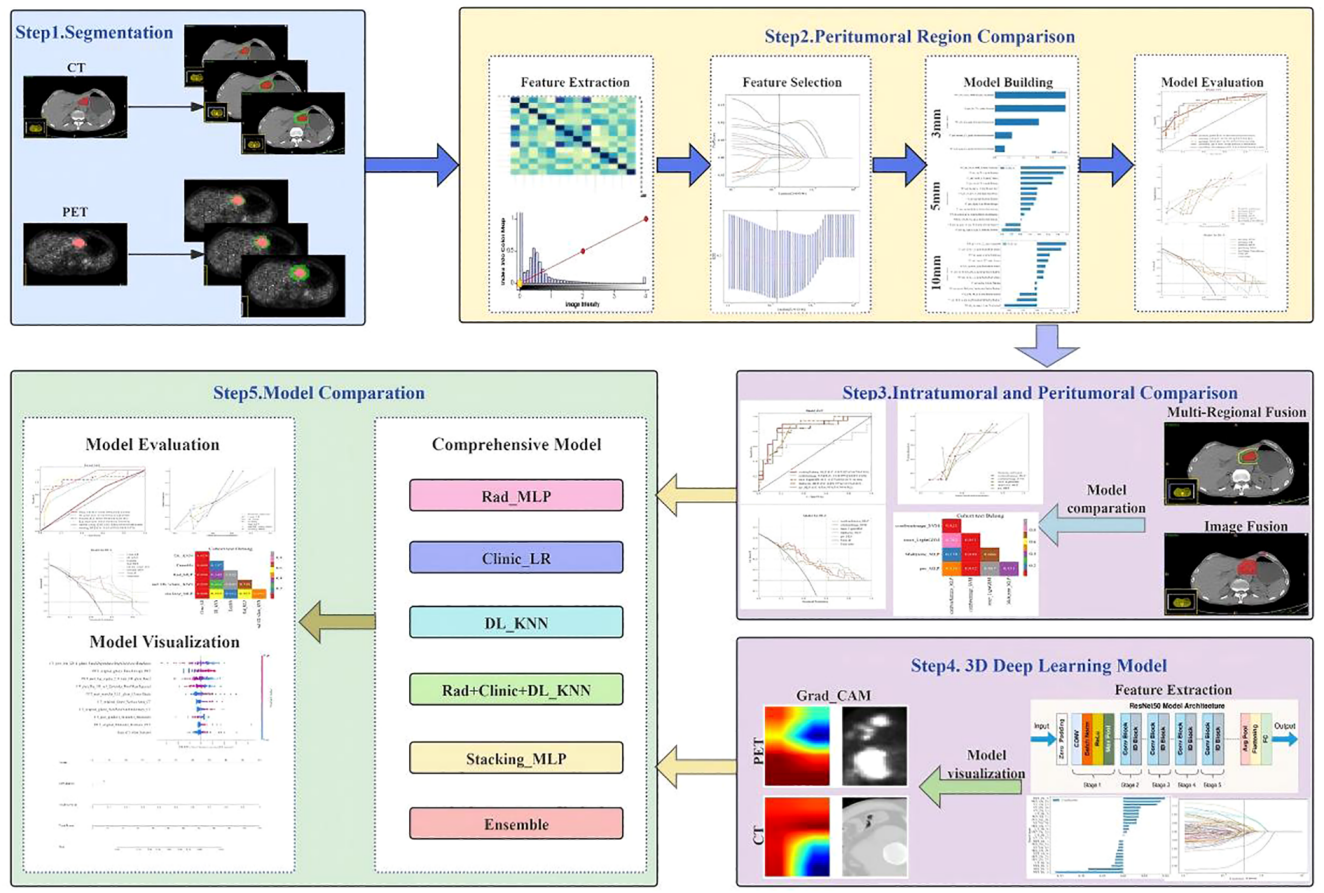
Overall workflow of the study. Manual segmentation of DLBCL lesions on PET/CT images was performed, followed by peritumoral expansion (3, 5, and 10 mm) and radiomic feature extraction. Feature selection was conducted using intraclass correlation coefficient (ICC), Spearman correlation, and LASSO. A deep learning model was built based on ResNet-50 transfer learning. Separate clinical, radiomic, and deep learning models were constructed, and then combined into three fusion models. The optimal model was subsequently subjected to SHAP-based interpretability analysis.

Radiomic features were extracted from all predefined regions of interest (ROIs) using the PyRadiomics (version 3.0.1) (23).Gray-level discretization was performed using a fixed bin count of 25. Intensity normalization was applied by centering at the mean ± 3 standard deviations and rescaling to [0,1]. Wavelet features were computed with the ‘coif1’ wavelet at a decomposition level of 1. Laplacian of Gaussian (LoG) features were extracted using sigma values of [1.0, 2.0, 3.0] mm. No local binary pattern (LBP) features were extracted. All feature definitions followed the Image Biomarker Standardisation Initiative (IBSI) guidelines (24). From CT images, 1,834 features were extracted, including 14 shape features, 360 first-order statistics, and 1,460 texture features. From PET images, 2,016 features were extracted, comprising 14 shape features, 396 first-order statistics, and 1,606 texture features. By combining intratumoral (IT) features with 3-mm, 5-mm, and 10-mm peritumoral features, 3,850 multi-scale features were generated. The entire dataset was randomly divided into a training set and an internal validation set at a 7:3 ratio. To eliminate the risk of data leakage, all preprocessing and feature selection procedures were performed exclusively on the training set: specifically, the mean and standard deviation for Z-score normalization were computed from the training set and subsequently applied to transform the internal validation set, and features with zero or near-zero variance were identified in the training set before being removed from both datasets. To reduce dimensionality and mitigate overfitting, a three-step feature selection pipeline was conducted solely using training data. First, highly correlated features (Spearman’s ρ > 0.9) were eliminated based on pairwise correlations derived from the training set to alleviate multicollinearity. Second, the least absolute shrinkage and selection operator (LASSO) with 10-fold cross-validation was implemented within the training set to retain features with non-zero coefficients, where the optimal penalty parameter λ was determined as the value that minimized cross-validated deviance (25). Third, the maximum relevance and minimum redundancy (mRMR) algorithm was applied to the training set to further select features with high predictive relevance and low inter-feature redundancy (26). The final feature sets selected for all candidate models are reported in **Supplementary Table 2**. Following feature engineering, all model selection procedures—including comparison of peritumoral distances, evaluation of candidate classifiers, and determination of the fusion strategy—were performed on the internal validation set. The training set was restricted to use for feature preprocessing, feature selection, and model fitting, while the internal validation set served to guide the final model architecture and hyperparameter selection. This analytical workflow ensured that only the most informative and non-redundant features were incorporated into model training, and the overall pipeline is illustrated in **Figure 3**.

Model development was conducted in two stages. In the first stage, to identify the most informative peritumoral region, single-region models were independently constructed using features from the Peri_3mm, Peri_5mm, and Peri_10mm regions. Each model was trained and evaluated using multiple machine learning classifiers, and the best-performing peritumoral region was defined as the “optimal peritumoral region.” In the second stage, to integrate intratumoral and peritumoral information, three different fusion strategies were designed and assessed (1): feature fusion, in which intratumoral and optimal peritumoral features were directly concatenated into a single feature vector prior to model training (2); image fusion, in which radiomic features were re-extracted from a combined mask encompassing both the intratumoral and optimal peritumoral regions (see **Figure 2**); and (3) multi-region fusion, incorporating all features from intratumoral (IT), Peri_3mm, Peri_5mm, and Peri_10mm regions to capture broader spatial metabolic heterogeneity. All fusion strategies were trained and validated using the same classifiers, and the model achieving the highest predictive accuracy and robustness was selected as the optimal conventional radiomics model.

### 3D deep learning model based on transfer learning

For each patient, the three-dimensional ROIs of the optimal intratumoral and peritumoral regions were cropped to their minimum bounding cubes to minimize background interference. Voxels were Z-score-normalized and resampled to an isotropic resolution. To enhance model robustness and prevent overfitting, real-time 3D data augmentation was applied during training, including random cropping, rotation, and axial flipping (27). In the validation set, only normalization and center cropping were performed to maintain analytical consistency and reproducibility.

The deep learning model employed a dual-branch 3D ResNet-50 architecture to independently extract features from PET and CT images. Specifically, PET and CT volumes were fed as separate single-channel inputs into two dedicated 3D ResNet-50 branches. To adapt the feature extractors to our task and leverage transfer learning, 2D ImageNet-pretrained weights were replicated along the third dimension to initialize each 3D branch—a widely adopted strategy for medical imaging tasks with limited training data. During fine-tuning, the original final fully-connected layer of each branch was temporarily replaced with a new classification layer containing two output nodes; after fine-tuning, these classification heads were discarded, and the 2048-dimensional features from the global average pooling layer of each branch were extracted and concatenated to form a fused feature vector (4096 dimensions). All input 3D ROIs were resized to 64 × 64 × 48 voxels. Model training used the Adam optimizer (initial learning rate 1e-3, batch size 4) for a maximum of 50 epochs, with a learning rate scheduler that reduced the rate by a factor of 0.5 when the internal validation loss plateaued for 5 consecutive epochs; early stopping with a patience of 10 epochs was also applied to mitigate overfitting. Binary cross-entropy loss was employed as the objective function. All experiments were implemented in the PyTorch framework, with random seeds fixed to ensure reproducibility. After extracting the fused deep features, we evaluated multiple classifiers to identify the optimal model for outcome prediction. Candidate classifiers included AdaBoost, Gradient Boosting, LightGBM, Logistic Regression, Multilayer Perceptron (MLP), Naive Bayes, Support Vector Machine (SVM), and K-Nearest Neighbors (KNN). Each classifier was trained on the fused features using the training set, and hyperparameters were optimized via grid search within the internal cross-validation framework on the training set. The best-performing classifier was selected based on cross-validated performance on the internal validation set, yielding the final model referred to as DL_KNN. Detailed comparison results are presented in the **Supplementary Table 3**.

### Model comparison and fusion strategies

To systematically evaluate the predictive value of different models for DEL, three baseline models were constructed. The clinical model was built from a comprehensive set of 13 candidate variables (including gender, age, involved site, IPI score, Ann Arbor stage, B symptoms, pathological subtype, Ki-67 index, β2-microglobulin, LDH, SUVmax, TLG, and MTV). The Hans IHC algorithm (CD10, BCL6, MUM1) was used to classify cases into GCB and non-GCB subtypes (28). After Z-score normalization, zero-variance filtering, Spearman correlation filtering, LASSO with 10-fold cross-validation, and mRMR—all performed strictly within the training set—only pathological subtype (GCB vs. non-GCB) was retained as the final predictor. The model was trained using a logistic regression classifier. The radiomics model was built based on the optimal conventional model selected in Section 5.3, representing a typical hand-crafted feature-based imaging approach. Intratumoral baseline model. PET and CT derived intratumoral radiomic features were fused, and five classifiers (LightGBM, LR, MLP, Naive Bayes, SVM) were trained on the fused feature set to construct the baseline intratumoral model with LightGBM selected as the final classifier, and the model is now consistently labeled as the Intratumoral model throughout the manuscript, **Supplementary Table 4**. The deep learning model (termed DL_KNN) employed a 3D ResNet-50 backbone as a feature extractor, taking standardized 3D ROI volumes as input. After fine-tuning, high-level semantic features were extracted from the global average pooling layer, and a K-Nearest Neighbors classifier was applied for final prediction (29). Based on these baselines, three multimodal fusion strategies were designed to integrate information across different levels (1): Feature-level fusion (Clinic+Rad+DL) concatenated radiomics features, deep learning features, and clinical variables into a single comprehensive feature vector, which was then used to train a downstream classifier; (2) Ensemble fusion (soft voting, Ensemble) averaged the predicted probabilities of the three baseline models to generate the final prediction, enhancing overall robustness; (3) Stacked ensemble fusion (Stacking) employed a two-layer meta-learning framework, where the first layer consisted of the three baseline models and the second layer was a logistic regression meta-classifier that used 10-fold cross-validated outputs from the first layer as input, achieving an optimally weighted combination and high-level information integration (30, 31).

### Model evaluation and selection

All models were constructed on the same training set and evaluated on the same internal validation set exclusively for exploratory model selection. The primary evaluation metric was the area under the ROC curve (AUC), along with accuracy, precision, recall, and F1 score. The model achieving the highest AUC was one of the top-performing candidate models and subsequently validated on an validation set. Additionally, the DeLong validation was used to assess the statistical significance of differences in AUC between the models. Decision curve analysis (DCA) was used to evaluate the clinical net benefit of the model. The clinical decision threshold was set at 0.42. Net benefit was calculated across threshold probabilities and compared with the “treat all” (IHC for all) and “treat none” (no IHC) strategies.

### Statistical analysis and computational environment

The normality of continuous clinical variables was assessed using the Shapiro–Wilk validation. Based on the distribution, continuous variables were compared using either the independent-samples t-validation or the Mann–Whitney U validation. The chi-square (χ²) validation was used to compare categorical variables. All data analyses were conducted on the OnekeyAI platform (version 4.9.1) using Python 3.7.12. Statistical evaluations were performed using the Statsmodels library (version 0.13.2). Radiomic feature extraction was performed using PyRadiomics (version 3.0.1). Machine learning algorithms, including support vector machine (SVM), were implemented using Scikit-learn (version 1.0.2). The deep learning framework was developed using PyTorch (version 1.11.0) and optimized with CUDA (version 11.3.1) and cuDNN (version 8.2.1) to enhance computational performance. The DeLong test was used to compare AUC differences between models. Net reclassification improvement (NRI) and integrated discrimination improvement (IDI) were calculated to quantify the incremental diagnostic value of the combined model relative to the clinical-only model. Subgroup analyses were performed to evaluate model stability across clinical subgroups; The 95% confidence intervals (CIs) for the area under the receiver operating characteristic curve (AUC) were calculated using the DeLong method in the main analyses, while 95% CIs for the area under the precision-recall curve (PR-AUC) were estimated via bootstrap resampling with 2000 iterations. For ablation experiments with limited sample size, 95% CIs for both AUC and PR-AUC were derived from bootstrap resampling with 2000 iterations. A two-sided P < 0.05 was considered statistically significant.

## Results

### Patient characteristics

This study included 143 patients with diffuse large B-cell lymphoma (DLBCL). In the training set, 36 patients (36.0%) were DEL-positive and 64 (64.0%) were DEL-negative; in the validation set, 19 patients (44.2%) were DEL-positive and 24 (55.8%) were DEL-negative. As summarized in **Table 1**, no statistically significant differences were observed between the training and validation sets in key clinical and imaging characteristics, including age, sex, IPI score, LDH level, and metabolic tumor volume (all p > 0.05), indicating a balanced data split and ensuring the validation set’s validity for evaluating model generalizability. Univariate and multivariate logistic regression analyses identified pathological type (GCB vs. non-GCB) as an independent predictor of DEL status (OR = 0.326, 95% CI: 0.157–0.676, p = 0.012), indicating that this subtype was a protective factor (**Table 2**). Accordingly, pathological type was incorporated as a key variable in the clinical model. The clinical model showed moderate discriminative ability, with an AUC of 0.607 (95% CI: 0.507–0.706) in the training set and 0.556 (95% CI: 0.414–0.698) in the validation set.

**Table 1:** Baseline clinical and imaging characteristics of patients with DLBCL.

Feature_name	Label=all	Label=validation	Label=train	P-value
ki67	76.5 ± 15.1	77.2 ± 12.7	76.2 ± 16.1	0.827
β_2MG	5.2 ± 17.9	3.8 ± 2.8	5.9 ± 21.3	0.677
LDH	351.3 ± 260.4	390.0 ± 335.4	334.7 ± 220.5	0.509
SUVmax	27.2 ± 12.8	28.1 ± 11.5	26.9 ± 13.4	0.314
TLG	5356.1 ± 6555.4	5414.2 ± 7685.0	5331.1 ± 6048.3	0.394
MTV	571.8 ± 744.9	554.6 ± 825.4	579.2 ± 711.8	0.386
Dmaxcm	31.7 ± 22.7	28.7 ± 23.2	33.0 ± 22.4	0.301
Gender				0.295
Female	61(42.7)	15(34.9)	46(46.0)	
Male	82(57.3)	28(65.1)	54(54.0)	
Age				0.5
≤60 years	72(50.4)	24(55.8)	48(48.0)	
>60 years	71(49.7)	19(44.2)	52(52.0)	
EOI				0.363
No	46(32.2)	11(25.6)	35(35.0)	
Yes	97(67.8)	32(74.4)	65(65.0)	
IPI				0.16
Score ≤ 2	72(50.4)	26(60.5)	46(46.0)	
Score>2	71(49.7)	17(39.5)	54(54.0)	
AnnArbor				0.116
I-II	48(33.6)	19(44.2)	29(29.0)	
III-IV	95(66.4)	24(55.8)	71(71.0)	
B_symptom				0.866
No	83(58.0)	24(55.8)	59(59.0)	
Yes	60(42.0)	19(44.2)	41(41.0)	
Pathological				0.157
Non-GCB	82(57.3)	29(67.4)	53(53.0)	
GCB	61(42.7)	14(32.6)	47(47.0)	

Lesion location (nodal/extranodal); Ann Arbor Stage (I-II: early-stage; III-IV: advanced-stage); IPI, International Prognostic Index (0-2: low-risk; 3-5: high-risk); Pathological Type (GCB, germinal center B-cell-like; non-GCB, non-germinal center B-cell-like subtype); Ki-67, Proliferation index; β2-MG, β2-microglobulin; LDH, Lactate dehydrogenase; SUVmax, Maximum standardized uptake value; TMTV, Total metabolic tumor volume; TLG, Total lesion glycolysis. *Significance level: p<0.05.

**Table 2:** Univariate and multivariate logistic regression analysis of clinical predictors for DEL.

Feature_name	Univariable analysis			Multivariable analysis		
	OR	95%CI	p_value	OR	95%CI	P_value
pathological	0.34	0.198-0.595	0.001	0.33	0.157-0.676	0.012
part	0.48	0.309-0.739	0.005	0.43	0.199-0.937	0.075
age	0.53	0.328-0.855	0.029	0.61	0.298-1.259	0.263
gender	0.64	0.402-1.007	0.105			
SUVmax	0.8	0.510-1.255	0.415			
AnnArbor	0.87	0.587-1.284	0.553			
B_symptom	0.95	0.569-1.592	0.876			
ki67	0.99	0.989-0.998	0.018	1.01	0.999-1.021	0.124
LDH	1	0.999-1.000	0.343			
β_2MG	1.01	0.986-1.035	0.486			
TLG	1.1	0.662-1.828	0.758			
IPI	1.25	0.797-1.962	0.415			
MTV	1.41	0.838-2.377	0.277			

Results of regression analysis identifying independent predictors of DEL. Pathological subtype was a significant protective factor (OR = 0.33, 95% CI: 0.157–0.676, p = 0.012).

### Performance of peritumoral models

**Figure 4:**
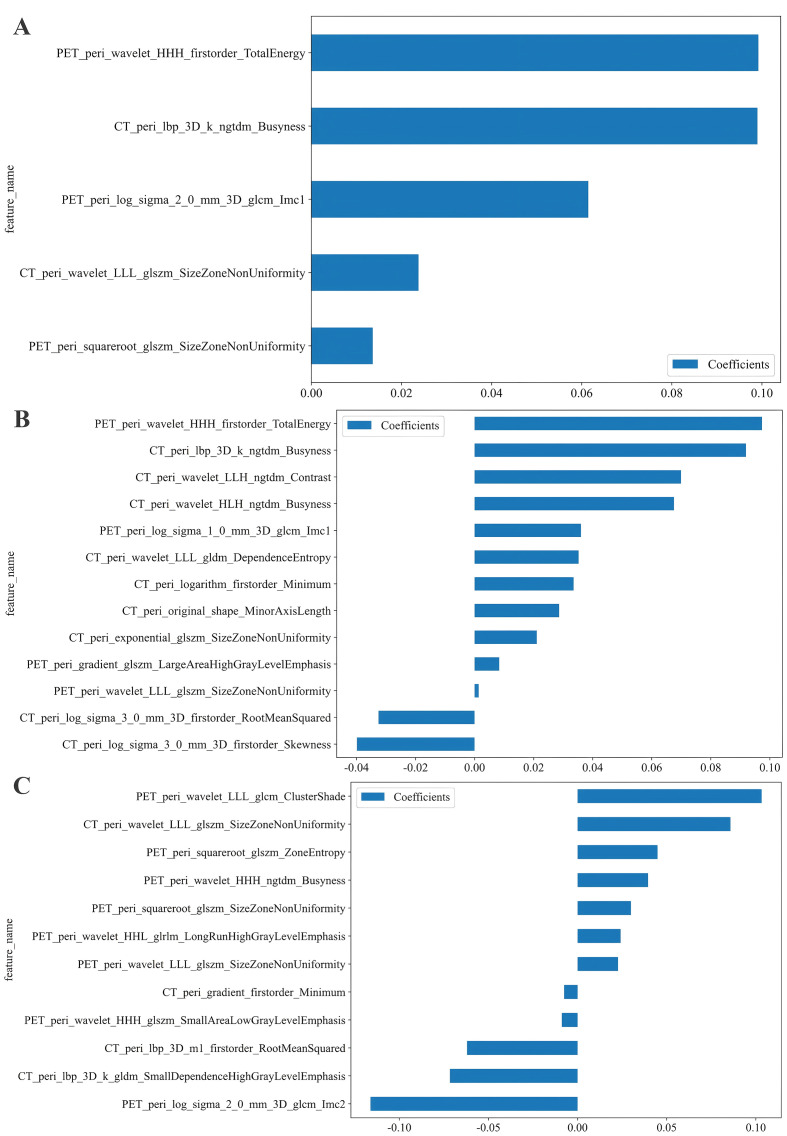
Feature selection and coefficients of peritumoral radiomics models. **(A–C)** Top-ranked features and corresponding coefficients for the Peri_3 mm, Peri_5 mm, and Peri_10 mm models, respectively. Redundant features were removed using Spearman correlation, followed by mRMR and LASSO for final selection. The Peri_10 mm model showed the most stable and predictive feature set, indicating that a wider peritumoral region captures more informative radiomic characteristics.

After removing redundant features based on Spearman correlation, a compact feature set was selected using the maximum relevance and minimum redundancy (mRMR) and LASSO algorithms. The Peri_3mm, Peri_5mm, and Peri_10mm models were subsequently trained with 5, 13, and 12 features, respectively, as shown in **Figure 4**. The predictive performance of radiomic models built from different peritumoral regions (3 mm, 5 mm, 10 mm) with various classifiers is detailed in **Table 3**. The results indicate that model performance strongly depends on both the spatial extent of the peritumoral region and the choice of classification algorithm. The model based on the 10-mm peritumoral region exhibited the best performance on the validation set. Specifically, the Peri_10mm_MLP model achieved an accuracy of 81.4% and an AUC of 0.836, followed closely by the Peri_10mm_NaiveBayes model with an AUC of 0.831. Both models demonstrated balanced diagnostic performance, with a sensitivity of 84.2%, a specificity of 79.2%, and an F1 score of 0.800. In contrast, models based on smaller peritumoral regions (3 mm, 5 mm) performed relatively poorly on the validation set, a trend consistent with the training-phase observations. As a targeted supplementary evaluation for the class-imbalanced dataset, precision-recall curves of all peritumoral models are provided in **Supplementary Figure 1**, with the average precision (AP) value of each model labeled in the legend. In the training cohort, the Peri10mm_MLP model achieved the highest AP value of 0.841, followed by Peri5mm_MLP (AP = 0.836), Peri5mm_LR (AP = 0.813), Peri10mm_NaiveBayes (AP = 0.757) and Peri3mm_KNN (AP = 0.719). In the validation cohort, the two 10-mm peritumoral models still maintained leading performance: Peri10mm_NaiveBayes reached an AP of 0.846 and Peri10mm_MLP reached an AP of 0.812, while the AP values of 3-mm and 5-mm peritumoral models ranged from 0.700 to 0.759, showing an obvious performance decline. The PR curve results are highly consistent with the ROC-based findings, further verifying that models built on the 10-mm peritumoral region achieve higher AP values and more stable recognition performance for DEL-positive samples. Consistent with the quantitative metrics, the confusion matrices (**Supplementary Figure 2**, panels B–F) visually show that peritumoral models with a 10-mm extent yield fewer misclassified samples than 3-mm and 5-mm peritumoral models.

**Table 3:** Performance comparison of peritumoral radiomics models.

Model	set	Accuracy	AUC (95% CI)	Sensitivity	Specificity	PPV	NPV	F1	PR AUC	Brier (95%CI)	Threshold
peri3mm_KNN	Train	0.75	0.849 (0.779 - 0.920)	0.78	0.73	0.62	0.86	0.69	0.719	0.149 (0.112 - 0.189)	0.40
	Validation	0.74	0.804 (0.676 - 0.932)	0.58	0.88	0.79	0.72	0.67	0.759	0.202 (0.126 - 0.295)	0.40
peri5mm_LR	Train	0.84	0.856 (0.780 - 0.932)	0.61	0.97	0.92	0.82	0.73	0.813	0.138 (0.101 - 0.180)	0.62
	Validation	0.72	0.726 (0.570 - 0.882)	0.68	0.75	0.68	0.75	0.68	0.700	0.234 (0.155 - 0.319)	0.28
peri5mm_MLP	Train	0.82	0.882 (0.815 - 0.949)	0.83	0.81	0.71	0.90	0.77	0.836	0.143 (0.113 - 0.175)	0.33
	Validation	0.77	0.763 (0.612 - 0.914)	0.79	0.75	0.71	0.82	0.75	0.756	0.203 (0.147 - 0.270)	0.29
peri10mm_MLP	Train	0.85	0.906 (0.850 - 0.962)	0.86	0.84	0.76	0.92	0.81	0.841	0.144 (0.115 - 0.193)	0.35
	Validation	0.81	0.836 (0.709 - 0.962)	0.84	0.79	0.76	0.86	0.80	0.812	0.179 (0.133 - 0.231)	0.35
peri10mm_NaiveBayes	Train	0.86	0.902 (0.843 - 0.961)	0.89	0.84	0.76	0.93	0.82	0.757	0.179 (0.111 - 0.166)	0.05
	Validation	0.81	0.831 (0.696 - 0.966)	0.84	0.79	0.76	0.86	0.80	0.846	0.212 (0.103 - 0.333)	0.032

AUC, area under the receiver operating characteristic curve; CI, confidence interval; PPV, positive predictive value; NPV, negative predictive value; PR AUC, area under the precision-recall curve; Brier, Brier score. The 95% CI for AUC was calculated using the DeLong method. The 95% CI for the Brier score was obtained via bootstrap resampling (2000 iterations). The optimal threshold for each model was determined by maximizing the Youden index in the training set and was subsequently applied to the validation set. Peri3mm, Peri5mm, and Peri10mm denote peritumoral regions with thicknesses of 3 mm, 5 mm, and 10 mm, respectively; KNN, k-nearest neighbors; LR, logistic regression; MLP, multilayer perceptron.

### Comparison of intratumoral and peritumoral models

**Figure 5:**
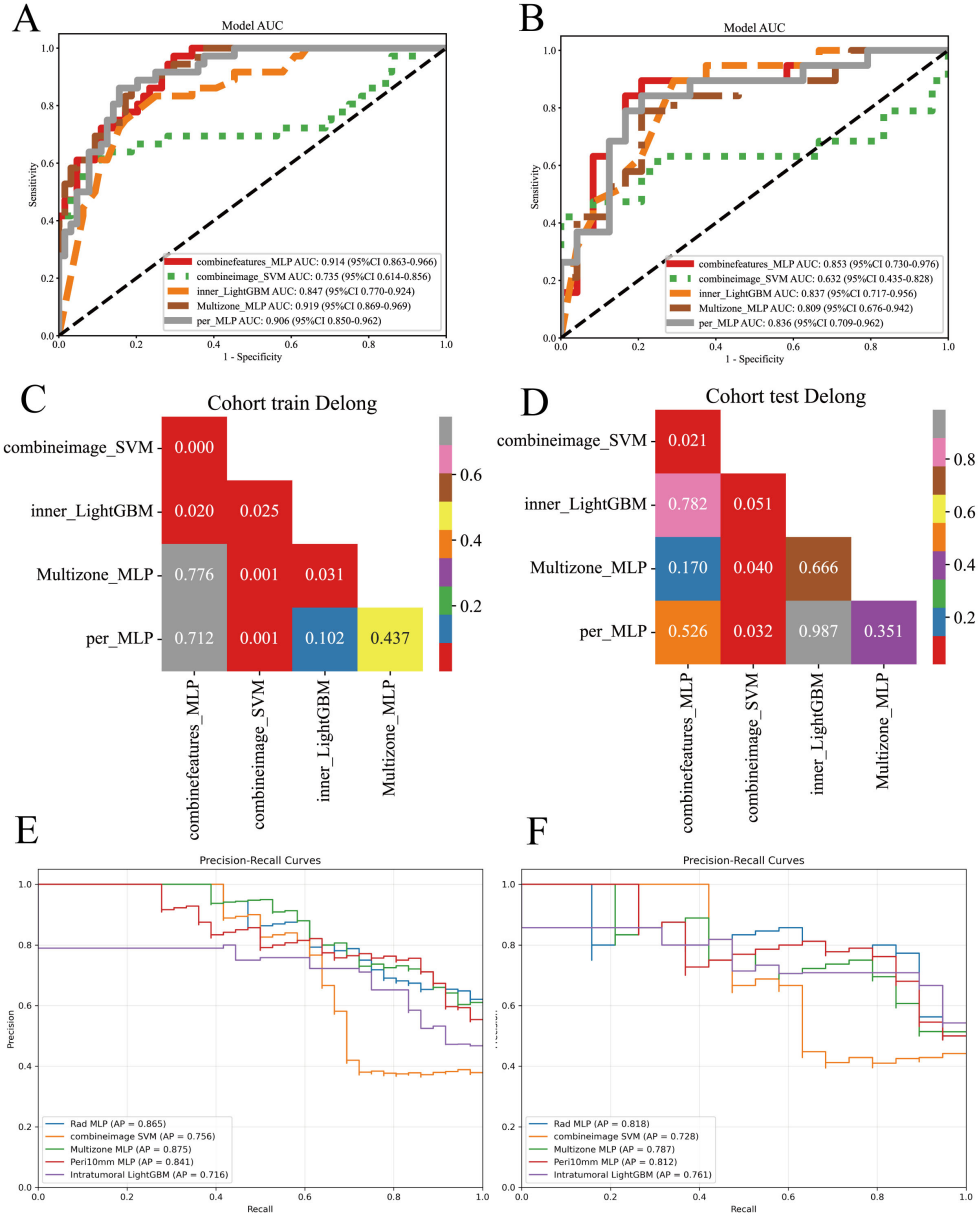
Comparison of intratumoral and peritumoral models for predicting DEL. **(A, B)** Receiver operating characteristic (ROC) curves of different radiomics models in the training and validationing cohorts. The combinefeatures_MLP models achieved the highest AUCs, indicating superior discriminative ability. **(C, D)** Pairwise DeLong validation heatmaps comparing AUCs among models in both cohorts. Color intensity represents p-values, where darker shades indicate greater statistical differences. Overall, the combinefeatures_MLP model demonstrated stable and robust performance across cohorts. **(E, F)** Precision-recall (PR) curves of multiple radiomic models in the training cohort **(E)** and validation cohort **(F)**. The x-axis represents recall and the y-axis represents precision. The average precision (AP) value of each model is labeled in the legend, which offers a more robust and informative performance assessment for imbalanced datasets.

Among the five classifiers trained on the fused intratumoral features, LightGBM achieved the highest and most stable performance on the validation set (AUC = 0.837), with no evidence of overfitting compared to the training set (AUC = 0.847). It was therefore selected as the classifier for the intratumoral baseline model. Full comparisons are provided in **Supplementary Table 4**. The diagnostic performance of radiomic models based on intratumoral, peritumoral and combined feature sets was evaluated in both the training and validation cohorts, with detailed quantitative metrics summarized in **Table 4** and comparative visualizations presented in **Figure 5**. The model integrating intratumoral and peritumoral features (Rad_MLP; combined features + multilayer perceptron) achieved the strongest discriminative capacity: it obtained an AUC of 0.914 (95% CI: 0.863–0.966) in the training cohort and the highest validation AUC of 0.853 (95% CI: 0.731–0.976), alongside favorable clinical performance with 0.90 sensitivity, 0.91 negative predictive value and 0.83 F1-score on the validation set. ROC curves, DeLong test-based pairwise statistical comparisons and precision-recall curves in **Figure 5** consistently corroborated the robust and balanced performance of the Rad_MLP model across multiple evaluation dimensions, confirming it as the optimal model for DEL status prediction. The full classification outcomes of all included models are visualized as confusion matrices in **Supplementary Figure 2**, which further verify the performance advantage of the multi-feature integrated model from the perspective of sample misclassification distribution.

**Table 4:** Performance of the Intratumoral and Peritumoral Models in the training and validation cohorts.

Model	Accuracy	AUC	95% CI	Sensitivity	Specificity	PPV	NPV	F1	Threshold	Brier score (95%CI)	PR AUC	Cohort
Rad_MLP	0.80	0.914	0.863 - 0.966	0.97	0.70	0.65	0.98	0.78	0.27	0.123 (0.094 - 0.153)	0.865	Train
combineimage_SVM	0.81	0.735	0.614 - 0.856	0.61	0.92	0.82	0.81	0.70	0.33	0.154 (0.115 - 0.193)	0.756	Train
intra_LightGBM	0.78	0.847	0.770 - 0.924	0.83	0.75	0.65	0.89	0.73	0.35	0.168 (0.139 - 0.199)	0.875	Train
Multizone_MLP	0.83	0.919	0.869 - 0.969	0.89	0.80	0.71	0.93	0.79	0.33	0.135 (0.109 - 0.166)	0.841	Train
per10mm_MLP	0.85	0.906	0.850 - 0.962	0.86	0.84	0.76	0.92	0.81	0.35	0.144 (0.115 - 0.175)	0.716	Train
Rad_MLP	0.84	0.853	0.731 - 0.976	0.90	0.79	0.77	0.91	0.83	0.28	0.176 (0.116 - 0.240)	0.818	Validation
combineimage_SVM	0.74	0.632	0.435 - 0.828	0.42	1.00	1.00	0.69	0.59	0.55	0.201 (0.141 - 0.271)	0.728	Validation
intra_LightGBM	0.79	0.837	0.717 - 0.956	0.90	0.71	0.71	0.90	0.79	0.33	0.195 (0.149 - 0.264)	0.761	Validation
Multizone_MLP	0.79	0.809	0.676 - 0.942	0.79	0.79	0.75	0.83	0.77	0.37	0.188 (0.140 - 0.242)	0.787	Validation
per10mm_MLP	0.81	0.836	0.709 - 0.962	0.84	0.79	0.76	0.86	0.80	0.35	0.179 (0.133 - 0.231)	0.812	Validation

AUC, area under the receiver operating characteristic curve; CI, confidence interval; PPV, positive predictive value; NPV, negative predictive value; PR AUC, area under the precision-recall curve. The 95% CI for AUC was calculated using the DeLong method. The 95% CI for the Brier score was obtained via bootstrap resampling (2000 iterations). The optimal threshold for each model was determined by maximizing the Youden index in the training set and was then applied to the validation set.

### Comprehensive model evaluation and selection

A comprehensive evaluation of various machine learning models was performed on both the training and validation sets. Key performance metrics, including accuracy, AUC, sensitivity, and specificity, are summarized in **Table 5**. Model performance was further validated in the validation set. Although some models showed a performance decline suggestive of potential overfitting to the training data, the Rad_MLP model maintained robust and balanced performance, achieving an accuracy of 83.7% and an AUC of 0.853. Rad_MLP demonstrated a strong balance between sensitivity (89.5%) and specificity (79.2%), with corresponding positive predictive value (PPV) and negative predictive value (NPV) of 0.773 and 0.905, respectively. The F1 score, reflecting the harmonic mean of precision and recall, was 0.829. The misclassification patterns of all models are visualized in **Supplementary Figure 3**, which intuitively corroborates the quantitative differences in **Table 5**. The stacking model achieved a slightly higher overall accuracy but presented more false-negative cases, while the Rad_MLP model showed the most balanced distribution between false positives and false negatives.

**Table 5:** Performance comparison of machine learning models for predicting DEL.

Model	set	Accuracy	AUC (95% CI)	Sensitivity	Specificity	PPV	NPV	F1	Threshold	Brier score (95%CI)	PR AUC
DL_KNN	Train	0.84	0.911 (0.859 - 0.963)	0.72	0.91	0.81	0.85	0.77	0.60	0.120 (0.096 - 0.146)	0.83
	Validation	0.70	0.774 (0.640 - 0.908)	0.68	0.71	0.65	0.74	0.67	0.40	0.199 (0.129 - 0.274)	0.68
Rad_MLP	Train	0.80	0.914 (0.863 - 0.966)	0.97	0.70	0.65	0.98	0.78	0.27	0.123 (0.094 - 0.153)	0.87
	Validation	0.84	0.853 (0.731 - 0.976)	0.90	0.79	0.77	0.91	0.83	0.28	0.176 (0.116 - 0.240)	0.82
Clinic_LR	Train	0.59	0.607 (0.507 - 0.706)	0.67	0.55	0.45	0.75	0.54	0.44	0.221 (0.192 - 0.249)	0.42
	Validation	0.54	0.556 (0.414 - 0.698)	0.74	0.38	0.48	0.64	0.58	0.44	0.247 (0.207 - 0.288)	0.47
Esamble	Train	0.92	0.973 (0.949 - 0.997)	0.94	0.91	0.85	0.97	0.90	0.38	0.121 (0.102 - 0.142)	0.96
	Validation	0.86	0.857 (0.728 - 0.987)	0.68	1.00	1.00	0.80	0.81	0.49	0.176 (0.130 - 0.229)	0.89
rad+DL+clinic_KNN	Train	0.89	0.956 (0.922 - 0.990)	0.86	0.91	0.84	0.92	0.85	0.60	0.108 (0.080 - 0.138)	0.85
	Validation	0.79	0.868 (0.768 - 0.969)	0.63	0.92	0.86	0.76	0.73	0.60	0.181 (0.116 - 0.259)	0.77
stacking_MLP	Train	0.90	0.969 (0.941 - 0.997)	0.97	0.86	0.80	0.98	0.88	0.30	0.097 (0.076 - 0.122)	0.95
	Validation	0.86	0.818 (0.658 - 0.978)	0.74	0.96	0.93	0.82	0.82	0.50	0.154 (0.102 - 0.218)	0.88

This table summarizes the diagnostic performance of six machine learning models with distinct feature combinations and classifiers, evaluated on the training cohort and independent validation cohort respectively. Abbreviations: DL_KNN, K-nearest neighbors classifier built on deep learning features; Rad_MLP, multilayer perceptron classifier integrating intratumoral and peritumoral radiomic features; Clinic_LR, logistic regression classifier based on clinical characteristics; Esamble, multi-source feature fusion ensemble model; rad+DL+clinic_KNN, K-nearest neighbors classifier combining radiomic, deep learning and clinical features; stacking_MLP, stacking ensemble model using multilayer perceptron as the meta-learner; AUC, area under the receiver operating characteristic curve; CI, confidence interval; PPV, positive predictive value; NPV, negative predictive value; PR AUC, area under the precision-recall curve.

In contrast, while the stacking_MLP and Ensemble models achieved comparable or slightly higher accuracies (86.0%), their performance trade-offs were less favorable for screening purposes. stacking_MLP showed high specificity (95.8%) but lower sensitivity (73.7%), whereas the Ensemble model achieved perfect specificity (1.000) and PPV (1.000) at the cost of substantially lower sensitivity (68.4%), which could result in an unacceptably high false-negative rate in clinical practice. Based on its consistent and balanced performance in the validation set—particularly its high sensitivity and robust AUC—the Rad_MLP model was selected as the optimal model for potential clinical application.

**Figure 6:**
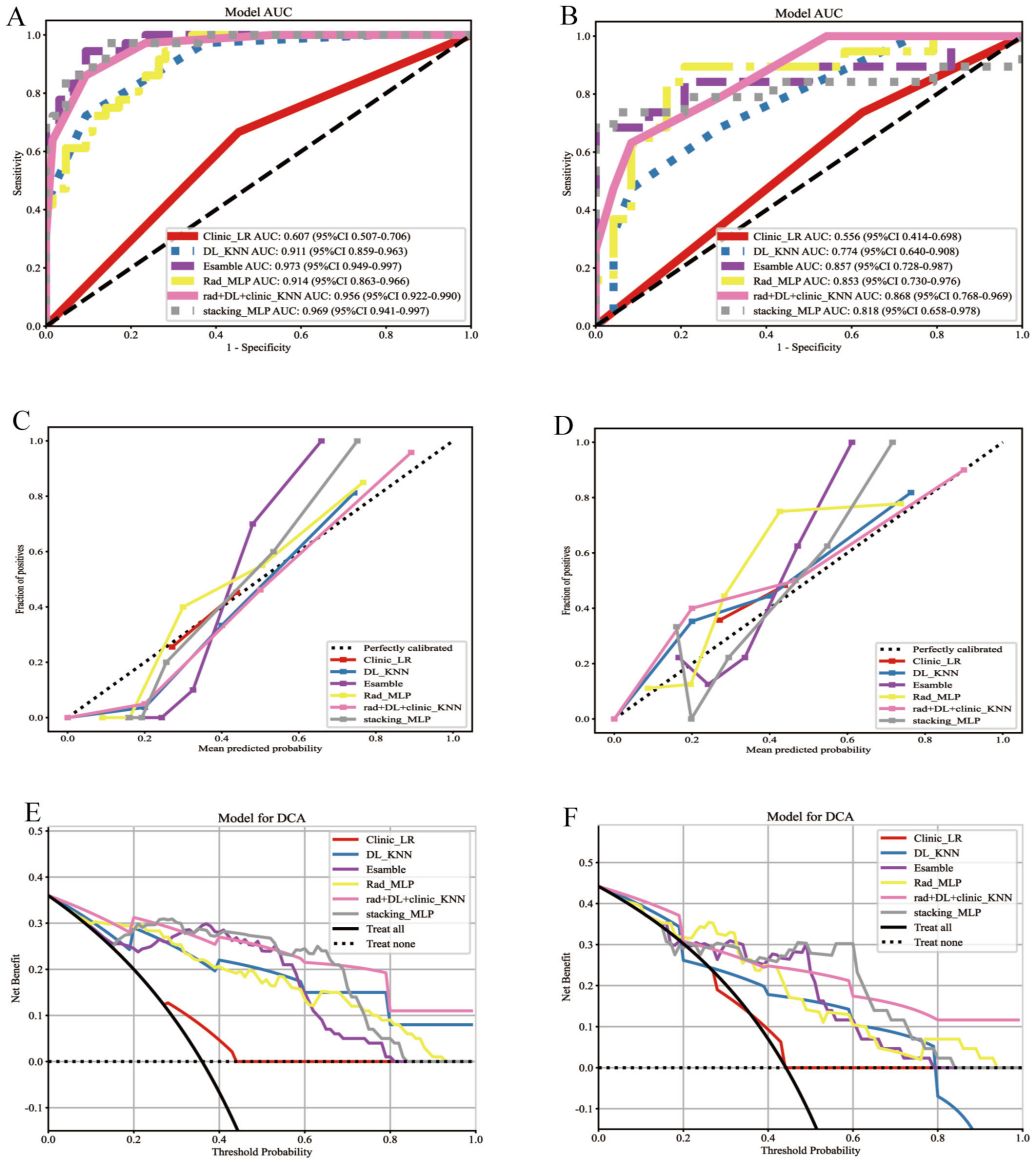
Comprehensive performance evaluation of machine learning models in predicting DEL. **(A, B)** Receiver operating characteristic (ROC) curves of multiple machine learning models in the training and validationing cohorts. The Rad_MLP model achieved the highest discriminative performance with an AUC of 0.853 (95% CI: 0.7305–0.9757), demonstrating robust generalizability and balanced sensitivity–specificity trade-off. **(C, D)** Calibration curves showing close agreement between predicted probabilities and observed outcomes in both the training and validationing cohorts, indicating well-calibrated probability estimates. **(E, F)** Decision curve analysis (DCA) demonstrating that the Rad_MLP model provides the highest net clinical benefit across a wide range of threshold probabilities, outperforming other classifiers and the “treat-all” or “treat-none” strategies.

**Figure 7:**
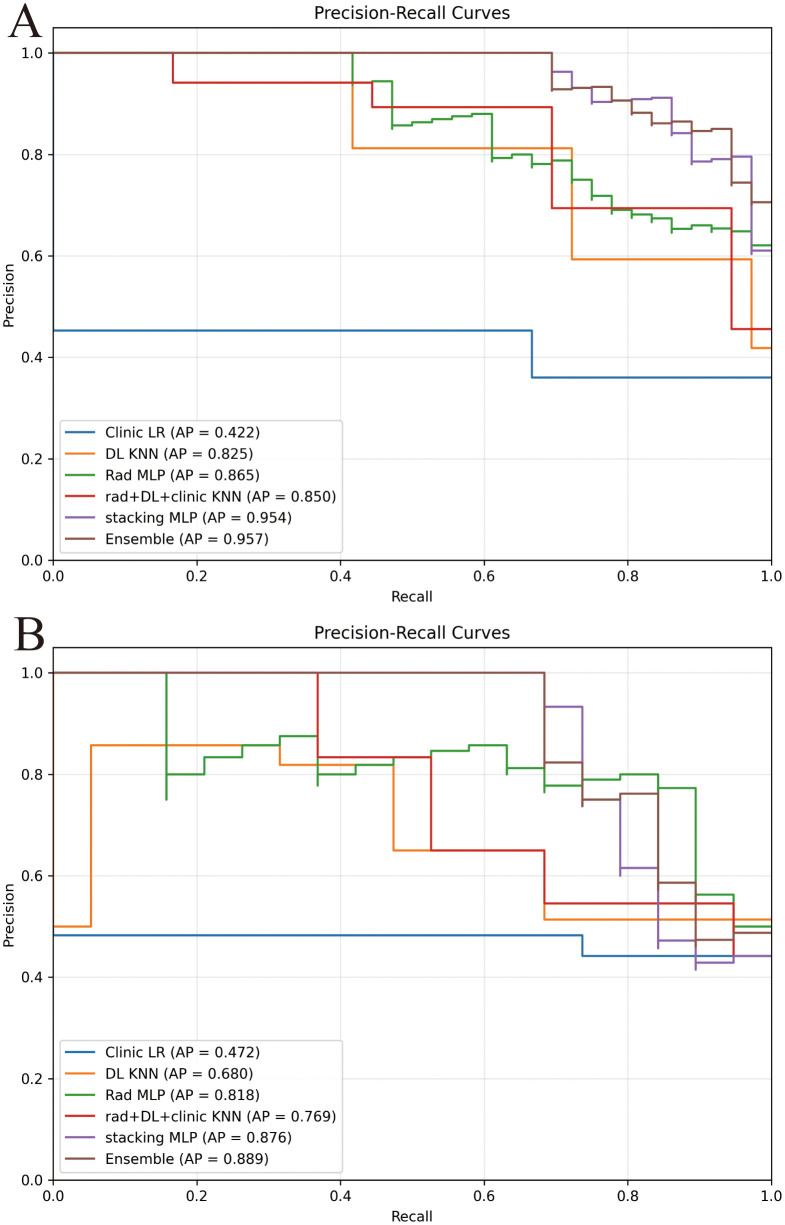
Precision-recall curves of models on the training and validation cohorts. **(A, B)** Precision-recall (PR) curves of all models in the training cohort **(A)** and validation cohort **(B)**. The x-axis represents recall and the y-axis represents precision. The average precision (AP) value, which corresponds to the area under the PR curve, is labeled for each model in the legend, serving as a robust complementary metric for model performance on imbalanced datasets.

The ROC curve for Rad_MLP confirmed its strong discriminative ability (AUC = 0.853). The calibration curve closely followed the ideal diagonal, indicating well-calibrated predicted probabilities that accurately reflect observed outcomes. Decision curve analysis (DCA) demonstrated that the Rad_MLP model provided a substantial net clinical benefit across a wide range of probability thresholds, as shown in **Figure 6**. At the pre-defined clinical decision threshold of 0.42, the model yielded a net benefit of approximately 0.165, which was superior to both the “treat all” and “treat none” strategies. The precision-recall curves are shown in **Figure 7**. Rad_MLP yielded APs of 0.865 on the training set and 0.818 on the test set, demonstrating performance close to the more complex ensemble (0.957/0.889) and stacking (0.954/0.876) models. Given that Rad_MLP relies solely on radiomics features and avoids deep-learning components, it was chosen as the final model for its better interpretability and lower risk of overfitting, with only a modest performance drop from training to test. To intuitively demonstrate the Rad_MLP misclassification patterns at the individual patient level, representative false-positive and false-negative cases are displayed in **Supplementary Figure 4**. The false-positive case (non-DEL patient PT54958) was incorrectly assigned a high DEL probability of 0.769, while the false-negative case (DEL patient PT53701) received an underestimated predicted probability of 0.174. These typical cases reflect the main error types of the model in practical prediction.

### Comprehensive model survey and ablation validation

Full performance metrics for all 15 candidate models are provided in **Supplementary Table 5**. The radiomics-only model (Rad_MLP) achieved high discrimination (AUC 0.853) and good calibration, with a Brier score of 0.176 and a non-significant Hosmer–Lemeshow test (P = 0.17; **Supplementary Table 6**). Notably, several complex fusion models exhibited poorer calibration (e.g., Ensemble model Hosmer–Lemeshow P < 0.001), indicating that additional features did not translate into more reliable probability estimates. Correspondingly, stepwise ablation on an independent test set (n=29, derived from a separate 6:2:2 random split; **Supplementary Table 7**) showed that removing clinical or DL features did not significantly degrade performance, and the radiomics-only configuration was not statistically inferior to the full ensemble (ΔAUC = −0.134, P = 0.096). Furthermore, formal incremental value testing confirmed that the combined model did not significantly improve discrimination over the clinical baseline in the validation set (IDI = 0.262, P = 0.211; NRI = 0.634) (**Supplementary Table 8**). Together, these results confirm that the simpler radiomics signature retains both robust discrimination and calibration without requiring additional inputs.

### Comparison with conventional PET metabolic parameters

We further compared the final radiomics model (Rad_MLP) against standard PET metabolic parameters. In both the training and validation cohorts, Rad_MLP consistently outperformed SUVmax, MTV, and their combination in discrimination and calibration (**Supplementary Table 9**).DeLong tests confirmed that these improvements were statistically significant in the training cohort (Rad_MLP vs. SUVmax, P = 0.044; vs. MTV, P < 0.001; vs. SUVmax+MTV+TLG, P = 0.021; **Supplementary Figure 5**). In the validation cohort, Rad_MLP maintained a numerically higher AUC (0.853; 95% CI, 0.731–0.976) with good calibration (Hosmer–Lemeshow P = 0.173), whereas SUVmax alone yielded a markedly lower AUC of 0.688 with poor calibration (P = 0.024); the AUC differences did not reach statistical significance for all comparisons (vs. SUVmax, P = 0.133; vs. MTV, P = 0.318; vs. SUVmax+MTV+TLG, P = 0.069), likely reflecting the limited validation sample size. These results indicate that the radiomics signature captures prognostic information beyond simplistic metabolic metrics.

### Subgroup performance of the final model

The robustness of Rad_MLP was assessed across key clinical subgroups in the internal validation cohort (**Supplementary Table 10**). The model maintained consistently good discrimination in most subgroups, including non-GCB subtype (AUC 0.924), extranodal disease (AUC 0.821), nodal disease (AUC 0.821), and Ann Arbor stage I–II (AUC 0.843) and III–IV (AUC 0.821). The GCB subgroup yielded a lower point estimate with a wide confidence interval (AUC 0.667, 95% CI 0.284–1.000), likely attributable to the small sample size (n = 14). Overall, these findings support the generalizability of the radiomics model across major disease presentations, while acknowledging the need for further validation in larger, balanced subgroups.

### Interpreting the optimal model using SHAP analysis

**Figure 8:**
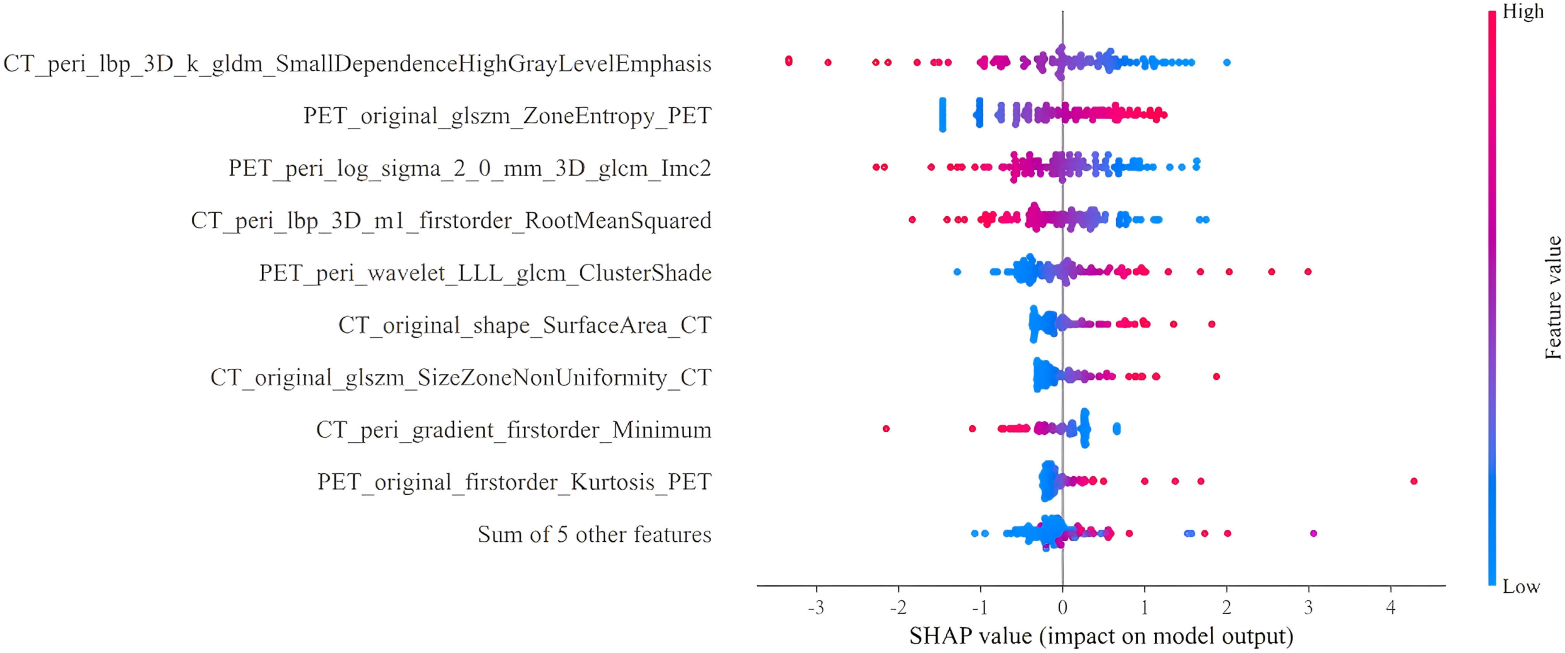
SHAP summary plot illustrating the feature importance and interpretability of the optimal Rad_MLP model. Site: The SHAP summary plot shows each feature’s contribution to model output. Each dot represents one patient, with color indicating feature value (red = high, blue = low).CT_peri_lbp_3D_k_gldm_SmallDependenceHighGrayLevelEmphasis and PET_original_glszm_ZoneEntropy_PET had the greavalidation positive SHAP values, indicating that increased peritumoral heterogeneity and complex gray-level patterns are associated with the DEL phenotype.

To explore the predictive mechanisms of the optimal Rad_MLP model, model interpretability was assessed using the SHAP (SHapley Additive exPlanations) framework. This approach quantitatively evaluates the contribution of each feature to the model output, thereby revealing key drivers of model decisions (32). As shown in **Figure 8**, SHAP analysis indicated that peritumoral features played a dominant role in model predictions, particularly high-order texture features such as CT_peri_lbp_3D_k_gldm_SmallDependenceHighGrayLevelEmphasis and PET_original_glszm_ZoneEntropy. High SHAP values for these features suggest that the complexity of gray-level distribution and texture heterogeneity in the peritumoral region are critical for distinguishing DEL from non-DEL cases. This finding is consistent with multiple radiomics studies indicating that peritumoral regions may potentially capture biological processes closely associated with tumor microenvironment alterations, including tumor invasiveness, immune cell infiltration, and stromal response (33). Moreover, the positive SHAP values of these texture features indicate that increased peritumoral heterogeneity is closely associated with the high aggressiveness of double-expressor lymphoma (DEL). These results further support the concept proposed by Aerts et al. (34) that radiomic features can serve as noninvasive quantitative biomarkers of tumor heterogeneity, a core driver of aggressive disease and poor prognosis. In summary, SHAP analysis not only validates the interpretability and biological plausibility of the optimal model but also provides a new noninvasive perspective for using radiomics to reveal DEL molecular characteristics and microenvironment heterogeneity.

## Discussion

In this study, we developed and validated a radiomic model that integrates intratumoral and peritumoral (10-mm expansion) features for the preoperative, noninvasive prediction of DEL. The model demonstrated strong performance on the validation set, achieving an AUC of 0.853, which indicates excellent discriminative ability and potential clinical utility.

Among the clinical variables evaluated, including age, sex, IPI score, LDH level, and metabolic tumor volume (MTV), only pathological subtype (GCB vs. non-GCB) emerged as an independent predictor of DEL status. Previous retrospective studies have suggested that the DEL subtype is associated with age, pathological type, LDH, and Ki-67 level (3), a finding that partially differs from our results. Nonetheless, non-GCB subtype, like DEL, generally exhibits higher aggressiveness and a poorer prognosis (35), which is consistent with the protective role of GCB subtype observed in our analysis. This discrepancy may stem from known biological differences in immune response, tumor microenvironment, and treatment sensitivity between GCB and non-GCB subtypes (36). Moreover, pathological classification may inherently capture the prognostic information carried by variables such as LDH and Ki-67; these factors could influence outcome through their interaction with pathological type, yet fail to reach independent statistical significance when examined in isolation.

We observed that features extracted from the 10-mm peritumoral region contributed substantially to model performance. This superiority likely reflects the ability of a wider margin to capture critical changes in the tumor microenvironment, a finding consistent with recent studies in lung cancer and brain tumors (37, 38). For other cancers, such as hepatocellular carcinoma and breast cancer, a 3-mm peritumoral region has been identified as optimal (39, 40). This difference may be explained by the particularly high aggressiveness, complex microenvironment, and immune evasion capacity of DEL, which may require a broader peritumoral sampling to adequately capture predictive biological signals.

With respect to feature-modeling strategies, the “intratumoral + 10-mm peritumoral” feature-fusion model outperformed both image-level fusion and multi-scale feature fusion. Several factors may account for this advantage. First, intratumoral features reflect the morphological and metabolic characteristics of the tumor core, whereas 10-mm peritumoral features capture microenvironmental attributes are hypothesized to reflect to immune infiltration and stromal remodeling. The complementary information provided by these two regions likely enhances prediction, in line with previous reports that integrating complementary feature sets improves diagnostic accuracy, such as in radiomics-based prediction of axillary lymph node metastasis (41). Second, image-level fusion may blur the distinction between core and peritumoral signals, whereas feature-level fusion of distinct regions generally yields better performance than single-region input (42). Finally, although multi-scale fusion incorporates hierarchical spatial information (e.g., 3-mm, 5-mm, and 10-mm regions), the resulting feature redundancy and accumulated noise may compromise model generalizability. Although DLBCL is a systemic hematologic malignancy, its involved lesions present as discrete tumor masses with a surrounding peritumoral zone characterized by immune cell infiltration, stromal reactions, and angiogenesis—features that may manifest as metabolic or structural alterations on PET/CT. Multiple studies have highlighted the prognostic significance of the immune infiltrate in DLBCL (43, 44), providing a biological rationale for incorporating peritumoral regions in our exploratory analysis. The expansion distances (3 mm, 5 mm, and 10 mm) were initially selected from solid-tumor radiomics literature; importantly, our multi-scale design was intended to systematically compare different expansion distances to empirically identify the most informative peritumoral feature set for our DLBCL cohort, rather than assuming *a priori* that any specific distance represents the true tumor microenvironment. Future studies with dedicated lymphoma-specific peritumoral annotation are warranted to further validate these findings.

In the comparison of integrated models, we found that the stacking_MLP and Ensemble models exhibited high specificity but relatively low sensitivity. This pattern illustrates the classic trade-off between sensitivity and specificity. In class-imbalanced settings, ensemble models tend to favor the majority class (negative samples), thereby missing a proportion of positive cases. This observation is consistent with Liu et al. (45), who noted that without dedicated class-weight adjustment or oversampling, ensemble learning models often struggle to balance these metrics. The low sensitivity of the stacking_MLP model may also result from insufficient learning from minority-class samples. These findings suggest that future studies should explore strategies such as class-weight adjustment, oversampling, or cost-sensitive learning to improve the detection of positive cases (46).We also explored deep learning models in this study; however, they did not achieve superior performance in our cohort, which may be attributable to the relatively limited sample size, and we consider this a direction for future research.

The series of supplementary analyses collectively reinforce the rationale behind our final model selection and its translational potential. Both the ablation study (**Supplementary Table 7**) and the formal incremental value assessment (**Supplementary Table 8**) converged to show that the inclusion of clinical and deep learning features did not significantly improve discrimination over the pure radiomics signature (ΔAUC P = 0.096; IDI P = 0.211 in the validation set), with the training-set gain failing to generalize—a pattern consistent with overfitting. Moreover, calibration evaluation revealed that the radiomics-only model maintained good calibration (Hosmer–Lemeshow P = 0.17), whereas several complex fusion models displayed significant miscalibration (**Supplementary Table 6**), further eroding the case for their adoption. These findings strongly support the adoption of a more parsimonious, imaging-only model. In addition, the radiomics model consistently and, in the training set, significantly outperformed conventional PET metabolic parameters (**Supplementary Table 9**, **Supplementary Figure 5**), indicating that high-dimensional texture and shape features capture prognostic information beyond simplistic metabolic metrics. Subgroup analysis further demonstrated the model’s robust performance across key clinical strata, including disease site and Ann Arbor stage, with AUCs consistently above 0.82 (**Supplementary Table 10**); the wider confidence interval in the small GCB subgroup (n = 14) simply underscores the need for larger validation in this subset. Taken together, these results confirm that the radiomics-only model not only preserves strong discrimination and good calibration without reliance on clinical variables, but also surpasses standard PET indices and maintains stability across heterogeneous disease presentations, thereby offering a reproducible and clinically pragmatic tool for individualized risk stratification.

To enhance model interpretability and foster clinical trust, we incorporated SHAP analysis, an explanation framework increasingly adopted in medical AI research. SHAP visualization identified key radiomic features that drive the model’s discrimination of DEL, particularly high-order texture features reflecting peritumoral heterogeneity and gray-level complexity. This suggests that irregular peritumoral image texture may correspond to more active immune responses and microenvironment remodeling (47). By moving from a “black-box” to a “white-box” approach, this interpretability strategy not only increases model transparency but also supports the clinical applicability of AI-based imaging tools.

Several limitations of this study should be acknowledged. As a single-center retrospective study validated only by an internal cohort, our findings are susceptible to the “winner’s curse” bias, and the generalizability of the proposed model to other patient populations remains to be further confirmed due to the absence of an independent external test set, which represents the major limitation of this work; meanwhile, the dataset was split via a single random partition, which may introduce sampling bias and cannot comprehensively evaluate the stability of the feature selection and model training procedures. Routine FISH testing for MYC, BCL2, and BCL6 rearrangements was not performed in this retrospective cohort; therefore, we cannot completely exclude the possibility that a small number of double-hit or triple-hit lymphoma cases may be present within the DEL group, although given the low overall incidence of DHL/THL and the consistency of the adverse prognostic features observed in our DEL cohort with prior published literature, any such confounding is unlikely to have substantially altered our main conclusions. Additionally, continuous quantitative data on MYC and BCL2 expression levels were not systematically collected, which precludes further validation of the direct biological correlation between the radiomic score and the molecular features of DEL. From the perspective of image preprocessing, all PET images were resampled to 1 × 1 × 1 mm³ isotropic voxels to enable accurate spatial expansion of peritumoral regions, but the interpolation applied during upsampling may intrinsically alter textural radiomic features, and formal sensitivity analyses with alternative voxel dimensions were not conducted within the scope of this exploratory study. Furthermore, the fixed peritumoral expansion distances (3, 5, and 10 mm) were adopted from solid tumor studies, and their biological plausibility may be limited in DLBCL, which often exhibits ill-defined margins and diffuse infiltration; no consensus currently exists on the optimal peritumoral expansion for lymphoma, so our peritumoral findings should be considered exploratory. Finally, no targeted validation via immunohistochemistry or gene expression analysis was performed to confirm the association between peritumoral regions and immune infiltration, stromal remodeling, and tumor microenvironment changes.

In summary, this study demonstrates the advantage of a radiomic model that integrates intratumoral and peritumoral features for DEL prediction and provides a biological rationale for this approach. Further work is warranted to address the current limitations and promote clinical translation. Large-scale multi-center prospective studies with independent external cohorts are needed to verify model robustness, mitigate the “winner’s curse” bias and confirm generalizability, with k-fold cross-validation adopted to improve algorithmic stability. Technically, sensitivity analyses of voxel sizes and lesion-adaptive peritumoral delineation with histological correlation will be explored to optimize the radiomic workflow. Additionally, systematic FISH testing, quantitative MYC/BCL2 measurement and multi-omics integration will be performed to elucidate the biological basis of the radiomic signature and its association with the tumor microenvironment, ultimately supporting the clinical application of radiomics for early diagnosis and personalized management of DEL.

## Conclusion

In this exploratory single-center study, we proposed and internally validated a Rad_MLP radiomic model that integrates intratumoral and 10 mm peritumoral features for the noninvasive prediction of double-expressor lymphoma (DEL; characterized by MYC/BCL2 co-expression) in DLBCL. The model demonstrated promising predictive performance, with an AUC of 0.853. SHAP analysis identified key peritumoral radiomic features driving the model’s decisions, offering potential biological insights. These findings suggest that a radiomics-based approach may serve as an exploratory tool for the noninvasive identification of DEL, pending external validation. If further validated in prospective multicenter cohorts, such a model could aid treatment stratification and prognostic assessment, and lay the groundwork for future multimodal and multi-omics integrative studies.

## Data Availability

The raw data supporting the conclusions of this article will be made available by the authors, without undue reservation.
