# Supplementary material for: Optimal peritumoral regions and fusion strategies for prediction of the double-expressor subtype in diffuse large B-cell lymphoma: a multi-region radiomics study
Source: Front Oncol. 2026 Jul 23;16:1877578. doi: 10.3389/fonc.2026.1877578 (PMC13442022; doi:10.3389/fonc.2026.1877578)
Supplement: Supplementary file 1 [file DataSheet1.docx]

| ID | group | label | ID | group | label | ID | group | label | ID | group | label |
| --- | --- | --- | --- | --- | --- | --- | --- | --- | --- | --- | --- |
| PT42624 | train | 0 | PT53346 | train | 0 | PT57558 | train | 1 | PT60732 | validation | 0 |
| PT45513 | train | 0 | PT53590 | train | 0 | PT57579 | train | 0 | PT60892 | train | 1 |
| PT46740 | validation | 0 | PT53627 | train | 0 | PT57972 | train | 0 | PT61223 | train | 0 |
| PT47135 | train | 1 | PT53701 | validation | 1 | PT58046 | train | 1 | PT61248 | train | 0 |
| PT47807 | train | 0 | PT53706 | validation | 0 | PT58213 | validation | 0 | PT61289 | train | 1 |
| PT48501 | train | 1 | PT53935 | validation | 0 | PT58216 | validation | 1 | PT61362 | validation | 0 |
| PT48650 | train | 0 | PT53936 | train | 0 | PT58238 | validation | 1 | PT61367 | validation | 0 |
| PT48815 | validation | 0 | PT54137 | train | 1 | PT58303 | train | 0 | PT61421 | validation | 0 |
| PT48964 | validation | 0 | PT54311 | train | 0 | PT58328 | train | 0 | PT61451 | train | 0 |
| PT49249 | train | 0 | PT54500 | train | 0 | PT58354 | train | 0 | PT61462 | train | 0 |
| PT49251 | validation | 0 | PT54502 | train | 0 | PT58386 | validation | 1 | PT61463 | validation | 0 |
| PT49411 | train | 1 | PT54512 | validation | 0 | PT58587 | train | 0 | PT61477 | train | 1 |
| PT49489 | train | 1 | PT54518 | train | 0 | PT58681 | train | 1 | PT61675 | train | 0 |
| PT49649 | train | 1 | PT54626 | train | 0 | PT58832 | validation | 1 | PT61692 | train | 0 |
| PT49666 | train | 1 | PT54851 | validation | 0 | PT58915 | validation | 1 | PT61715 | validation | 1 |
| PT49829 | train | 0 | PT54889 | train | 0 | PT58979 | validation | 0 | PT61717 | train | 0 |
| PT50110 | validation | 0 | PT54958 | validation | 0 | PT59066 | validation | 1 | PT61756 | train | 0 |
| PT50176 | train | 0 | PT54996 | train | 0 | PT59079 | train | 1 | PT61757 | train | 0 |
| PT50182 | validation | 1 | PT55453 | train | 0 | PT59130 | train | 0 | PT61795 | train | 1 |
| PT50793 | train | 0 | PT55492 | train | 0 | PT59263 | train | 1 | PT61923 | validation | 1 |
| PT50831 | train | 1 | PT55606 | validation | 0 | PT59304 | train | 0 | PT62099 | validation | 0 |
| PT50936 | train | 0 | PT55670 | train | 1 | PT59311 | validation | 1 | PT62107 | train | 1 |
| PT51139 | validation | 1 | PT55933 | validation | 1 | PT59370 | train | 0 | PT62259 | train | 1 |
| PT51360 | train | 0 | PT55950 | validation | 1 | PT59411 | train | 1 |  |  |  |
| PT51495 | validation | 0 | PT56044 | train | 0 | PT59542 | train | 1 |  |  |  |
| PT51760 | train | 1 | PT56051 | train | 0 | PT59644 | train | 1 |  |  |  |
| PT51982 | validation | 0 | PT56148 | train | 1 | PT59657 | validation | 1 |  |  |  |
| PT52093 | validation | 0 | PT56544 | train | 0 | PT59707 | train | 0 |  |  |  |
| PT52173 | train | 1 | PT56551 | train | 1 | PT59711 | validation | 0 |  |  |  |
| PT52258 | train | 0 | PT56560 | train | 0 | PT59811 | train | 1 |  |  |  |
| PT52282 | train | 0 | PT56577 | train | 0 | PT59829 | validation | 1 |  |  |  |
| PT52310 | train | 0 | PT56871 | train | 1 | PT59917 | train | 1 |  |  |  |
| PT52319 | train | 1 | PT56896 | train | 0 | PT60030 | train | 0 |  |  |  |
| PT52474 | validation | 0 | PT57030 | train | 0 | PT60115 | validation | 1 |  |  |  |
| PT52597 | train | 1 | PT57042 | train | 0 | PT60296 | train | 0 |  |  |  |
| PT52634 | train | 0 | PT57119 | train | 0 | PT60298 | train | 1 |  |  |  |
| PT52680 | train | 0 | PT57295 | train | 1 | PT60309 | train | 1 |  |  |  |
| PT52732 | train | 0 | PT57311 | train | 0 | PT60432 | train | 0 |  |  |  |
| PT52852 | train | 0 | PT57451 | train | 0 | PT60600 | validation | 1 |  |  |  |
| PT53221 | train | 1 | PT57475 | train | 0 | PT60684 | validation | 1 |  |  |  |

Supplementary Table 1. Patient ID list, cohort assignment and class labels for the 7:3 stratified train–validation split

Note. This table summarizes patient IDs and their 7:3 stratified split into training and internal validation cohorts. The random seed for data splitting is 42. Label 0 and 1 represent non-DEL and DEL respectively.

Supplementary Table 2. Final selected features for each candidate prediction model

| Model | feature_name |
| --- | --- |
| Intra_LightGBM | CT_original_shape_SurfaceArea_CT |
|  | PET_original_firstorder_Kurtosis_PET |
|  | CT_original_glrlm_ShortRunLowGrayLevelEmphasis_CT |
|  | PET_original_glszm_ZoneEntropy_PET |
|  | CT_original_glszm_SizeZoneNonUniformity_CT |
| peri3mm_Rad | PET_peri_wavelet_HHH_firstorder_TotalEnergy |
|  | CT_peri_lbp_3D_k_ngtdm_Busyness |
|  | PET_peri_log_sigma_2_0_mm_3D_glcm_Imc1 |
|  | CT_peri_wavelet_LLL_glszm_SizeZoneNonUniformity |
|  | PET_peri_squareroot_glszm_SizeZoneNonUniformity |
| peri5mm_Rad | PET_peri_wavelet_HHH_firstorder_TotalEnergy |
|  | CT_peri_lbp_3D_k_ngtdm_Busyness |
|  | CT_peri_wavelet_LLH_ngtdm_Contrast |
|  | CT_peri_wavelet_HLH_ngtdm_Busyness |
|  | PET_peri_log_sigma_1_0_mm_3D_glcm_Imc1 |
|  | CTperi_wavelet_LLL_gldm_DependenceEntropy |
|  | CT_peri_logarithm_firstorder_Minimum |
|  | CT_peri_original_shape_MinorAxisLength |
|  | CT_peri_exponential_glszm_SizeZoneNonUniformity |
|  | PET_peri_gradient_glszm_LargeAreaHighGrayLevelEmphasis |
|  | PET_peri_wavelet_LLL_glszm_SizeZoneNonUniformity |
|  | CT_peri_log_sigma_3_O_mm_3D_firstorder_RootMeanSquared |
|  | CT_peri_log_sigma_3_O_mm_3D_firstorder_Skewness |
| peri10mm_Rad | PET_peri_wavelet_LLL_glcm_ClusterShade |
|  | CT_peri_wavelet_LLL_glszm_SizeZoneNonUniformity |
|  | PET_peri_squareroot_glszm_ZoneEntropy |
|  | PET_peri_wavelet_HHH_ngtdm_Busyness |
|  | PET_peri_squareroot_glszm_SizeZoneNonUniformity |
|  | PET_peri_wavelet_HHL_glrlm_LongRunHighGrayLevelEmphasis |
|  | PET_peri_wavelet_LLL_glszm_SizeZoneNonUniformity |
|  | CT_peri_gradient_firstorder_Minimum |
|  | PET_peri_wavelet_HHH_glszm_SmallAreaLowGrayLevelEmphasis |
|  | CT_peri_lbp_3D_ml_firstorder_RootMeanSquared |
|  | CT_peri_lbp_3D_k_gldm_SmallDependenceHighGrayLevelEmphasis |
|  | PET_peri_log_sigma_2_0_mm_3D_glcm_Imc2 |
| combinefeatures_MLP | CT_original_glszm_SizeZoneNonUniformity_CT |
|  | PET_peri_wavelet_LLL_glcm_ClusterShade |
|  | PET_peri_squareroot_glszm_SizeZoneNonUniformity |
|  | PET_peri_squareroot_glszm_ZoneEntropy |
|  | CT_peri_wavelet_LLL_glszm_SizeZoneNonUniformity |
|  | PET_original_glszm_ZoneEntropy_PET |
|  | PET_original_firstorder_Kurtosis_PET |
|  | PET_original_firstorder_TotalEnergy_PET |
|  | CT_original_glrlm_ShortRunLowGrayLevelEmphasis_CT |
|  | CT_original_firstorder_Skewness_CT |
|  | CT_peri_lbp_3D_m1_firstorder_RootMeanSquared |
|  | CT_peri_lbp_3D_k_gldm_SmallDependenceHighGrayLevelEmphasis |
|  | PET_peri_log_sigma_2_0_mm_3D_glcm_Imc2 |
| combineimage_SVM | CT_intra_original_glszm_SizeZoneNonUniformity |
|  | PET_intra_original_glszm_LargeAreaLowGrayLevelEmphasis |
|  | CT_intra_original_shape_VoxelVolume |
|  | PET_intra_original_glrlm_ShortRunLowGrayLevelEmphasis |
| Multizone_MLP | PET_peri5mm_wavelet_HHH_firstorder_TotalEnergy |
|  | CT_original_glszm_SizeZoneNonUniformity_CT |
|  | CT_peri5mm_lbp_3D_k_ngtdm_Busyness |
|  | PET_peri1Omm_squareroot_glszm_ZoneEntropy |
|  | PET_peri10mm_wavelet_LLL_glcm_ClusterShade |
|  | PET_original_firstorder_Kurtosis_PET |
|  | CT_original_glrlm_ShortRunLowGrayLevelEmphasis_CT |
|  | CT_peri1Omm_lbp_3D_m1_firstorder_RootMeanSquared |
|  | CT_peri10mm_lbp_3Dk_gldm_SmallDependenceHighGrayLevelEmphasis |
|  | PET_peri10mm_log_sigma_2_0_mm_3D_glcm_Imc2 |
| DL-KNN | PET_DL_8 |
|  | PET_DL_19 |
|  | CT_DL_27 |
|  | CT_DL_16 |
|  | CT_DL_11 |
|  | CT_DL_9 |
|  | PET_DL_11 |
|  | PET_DL_20 |
|  | CT_DL_30 |
|  | PET_DL_17 |
|  | CT_DL_6 |
|  | PET_DL_25 |
|  | CT_DL_2 |
|  | CT_DL_17 |
|  | PET_DL_1 |
|  | PET_DL_14 |
|  | CT_DL_10 |
|  | PET_DL_26 |
|  | PET_DL_0 |
|  | PET_DL_31 |
|  | PET_DL_27 |
|  | CT_DL_8 |
|  | PET_DL_6 |
|  | PET_DL_5 |
|  | PET_DL_2 |
| Clinic_LR | Pathological |
| rad+clinic+DL_KNN | PET_DL_19 |
|  | CT_DL_27 |
|  | CT_original_glszm_SizeZoneNonUniformity_CT |
|  | PET_DL_8 |
|  | PET_peri_squareroot_glszm_ZoneEntropy |
|  | CT_DL_30 |
|  | PET_original_firstorder_TotalEnergy_PET |
|  | PET_DL_11 |
|  | PET_peri_wavelet_LLL_glcm_ClusterShade |
|  | CT_original_firstorder_Skewness_CT |
|  | CT_DL_6 |
|  | CT_DL_16 |
|  | PET_original_firstorder_Skewness_PET |
|  | PET_DL_25 |
|  | CT_DL_9 |
|  | PET_DL_17 |
|  | PET_peri_wavelet_HHH_ngtdm_Busyness |
|  | CT_DL_2 |
|  | CT_original_glrlm_ShortRunLowGrayLevelEmphasis_CT |
|  | CT_DL_11 |
|  | CT_original_firstorder_90Percentile_CT |
|  | CT_original_glrlm_RunEntropy_CT |
|  | CT_DL_10 |
|  | CT_original_shape_MajorAxisLength_CT |
|  | PET_DL_0 |
|  | PET_DL_6 |
|  | PET_DL_26 |
|  | PET_original_glcm_MCC_PET |
|  | CT_peri_lbp_3D_k_gldm_SmallDependenceHighGrayLevelEmphasis |
|  | PET_peri_1og_sigma_2_0_mm_3D_glcm_Imc2 |
|  | PET_DL_5 |
|  | CT_peri_lbp_3D_m1_firstorder_RootMeanSquared |
|  | PET_DL_2 |
| stacking_MLP | DL_label_1 |
|  | Rad_label_1 |
|  | Clinic_label_1 |

All features were selected via least absolute shrinkage and selection operator (LASSO) regression in the training cohort

Note. This table lists the final selected features for each prediction model, covering radiomic, deep learning-derived and clinical features. Abbreviations: Intra, intratumoral; Peri, peritumoral; Rad, radiomics; DL, deep learning; Clinic, clinical; LightGBM, light gradient boosting machine; KNN, k-nearest neighbor; LR, logistic regression; MLP, multilayer perceptron; SVM, support vector machine.

Supplementary Table 3.Diagnostic performance of machine learning classifiers with 3D ResNet-50-derived deep features in training and validation cohorts

| Model | Accuracy | AUC | 95% CI | Sensitivity | Specificity | F1 | Threshold | Cohort |
| --- | --- | --- | --- | --- | --- | --- | --- | --- |
| AdaBoost | 0.97 | 0.994 | 0.985 - 1.000 | 0.94 | 0.98 | 0.96 | 0.49 | Train |
| GradientBoosting | 0.96 | 0.997 | 0.991 - 1.000 | 1.00 | 0.94 | 0.95 | 0.37 | Train |
| KNN | 0.84 | 0.911 | 0.859 - 0.963 | 0.72 | 0.91 | 0.77 | 0.60 | Train |
| LightGBM | 0.85 | 0.939 | 0.898 - 0.981 | 0.92 | 0.81 | 0.82 | 0.35 | Train |
| LR | 0.98 | 0.994 | 0.984 - 1.000 | 1.00 | 0.97 | 0.97 | 0.41 | Train |
| MLP | 0.96 | 0.990 | 0.977 - 1.000 | 0.97 | 0.95 | 0.95 | 0.40 | Train |
| NaiveBayes | 0.89 | 0.911 | 0.850 - 0.972 | 0.75 | 0.97 | 0.83 | 0.91 | Train |
| SVM | 0.98 | 0.995 | 0.987 - 1.000 | 1.00 | 0.97 | 0.97 | 0.38 | Train |
| AdaBoost | 0.81 | 0.814 | 0.672 - 0.956 | 0.79 | 0.83 | 0.79 | 0.48 | Validation |
| GradientBoosting | 0.74 | 0.760 | 0.610 - 0.910 | 0.63 | 0.83 | 0.69 | 0.51 | Validation |
| KNN | 0.70 | 0.774 | 0.640 - 0.908 | 0.68 | 0.71 | 0.67 | 0.40 | Validation |
| LightGBM | 0.74 | 0.780 | 0.640 - 0.919 | 0.84 | 0.67 | 0.74 | 0.33 | Validation |
| LR | 0.77 | 0.770 | 0.620 - 0.920 | 0.68 | 0.83 | 0.72 | 0.43 | Validation |
| MLP | 0.79 | 0.800 | 0.661 - 0.940 | 0.68 | 0.88 | 0.74 | 0.36 | Validation |
| NaiveBayes | 0.72 | 0.671 | 0.498 - 0.844 | 0.53 | 0.88 | 0.63 | 0.80 | Validation |
| SVM | 0.79 | 0.787 | 0.642 - 0.933 | 0.63 | 0.92 | 0.73 | 0.42 | Validation |

Note. Abbreviations: 3D ResNet-50, 3D residual network with 50 layers; CNN, convolutional neural network; AUC, area under the receiver operating characteristic curve; 95% CI, 95% confidence interval of AUC; F1, F1-score; AdaBoost, adaptive boosting; KNN, k-nearest neighbor; LightGBM, light gradient boosting machine; LR, logistic regression; MLP, multilayer perceptron; SVM, support vector machine. All classifiers were built on deep features extracted by the 3D ResNet-50 model. The optimal classification threshold was determined by maximizing Youden’s index on the training set.

Supplementary Table 4. Diagnostic performance of different classifiers for the intratumoral baseline radiomics model based on fused PET and CT intratumoral features in the training and internal validation cohorts

| Model | Accuracy | AUC | 95% CI | Sensitivity | Specificity | PPV | NPV | Precision | Recall | F1 | Threshold | Cohort |
| --- | --- | --- | --- | --- | --- | --- | --- | --- | --- | --- | --- | --- |
| LightGBM | 0.78 | 0.847 | 0.770 - 0.924 | 0.83 | 0.75 | 0.65 | 0.89 | 0.65 | 0.83 | 0.73 | 0.35 | Train |
| LR | 0.80 | 0.839 | 0.753 - 0.925 | 0.75 | 0.83 | 0.71 | 0.86 | 0.71 | 0.75 | 0.73 | 0.35 | Train |
| MLP | 0.81 | 0.841 | 0.758 - 0.923 | 0.72 | 0.86 | 0.74 | 0.85 | 0.74 | 0.72 | 0.73 | 0.38 | Train |
| NaiveBayes | 0.80 | 0.818 | 0.727 - 0.909 | 0.64 | 0.89 | 0.77 | 0.81 | 0.77 | 0.64 | 0.70 | 0.22 | Train |
| SVM | 0.77 | 0.852 | 0.769 - 0.934 | 0.83 | 0.73 | 0.64 | 0.89 | 0.64 | 0.83 | 0.72 | 0.26 | Train |
| LightGBMd | 0.79 | 0.837 | 0.717 - 0.956 | 0.90 | 0.71 | 0.71 | 0.90 | 0.71 | 0.90 | 0.79 | 0.33 | Validation |
| LR | 0.74 | 0.811 | 0.676 - 0.947 | 0.90 | 0.63 | 0.65 | 0.88 | 0.65 | 0.90 | 0.76 | 0.20 | Validation |
| MLP | 0.79 | 0.836 | 0.707 - 0.964 | 0.74 | 0.83 | 0.78 | 0.80 | 0.78 | 0.74 | 0.76 | 0.36 | Validation |
| NaiveBayes | 0.72 | 0.781 | 0.644 - 0.917 | 0.90 | 0.58 | 0.63 | 0.88 | 0.63 | 0.90 | 0.74 | 0.03 | Validation |
| SVM | 0.70 | 0.757 | 0.609 - 0.904 | 0.95 | 0.50 | 0.60 | 0.92 | 0.60 | 0.95 | 0.74 | 0.24 | Validation |

Note. This intratumoral baseline model was constructed using fused intratumoral radiomic features extracted from PET and CT images. Five machine learning classifiers were trained and evaluated, with LightGBM selected as the final classifier for the intratumoral model throughout the manuscript. Abbreviations: AUC, area under the receiver operating characteristic curve; 95% CI, 95% confidence interval of AUC; PPV, positive predictive value; NPV, negative predictive value; F1, F1-score; Rad, radiomics; LightGBM, light gradient boosting machine; LR, logistic regression; MLP, multilayer perceptron; SVM, support vector machine. The optimal classification threshold was determined by maximizing Youden’s index in the training cohort and then applied to the internal validation cohort.

Supplementary Figure 1. Precision-recall (PR) curves of peritumoral radiomic models constructed with different peritumoral margin widths (3 mm, 5 mm, 10 mm) and multiple classifiers.

(A) PR curves of all peritumoral models in the training cohort. (B) PR curves of all peritumoral models in the validation cohort. The average precision (AP) value, equivalent to the area under the PR curve, is provided for each model in the legend. As a robust complementary metric to ROC-AUC, PR curves are particularly suitable for performance evaluation on class-imbalanced datasets.

Supplementary Figure 2.Confusion matrices showing the classification performance of nine radiomic models with distinct feature sets and classifiers for DEL status prediction in the validation cohort

Panels A–I correspond to the following models: (A) intratumoral radiomic model built with the LightGBM classifier; (B) 3-mm peritumoral radiomic model built with the KNN classifier; (C) 5-mm peritumoral radiomic model built with the LR classifier; (D) 5-mm peritumoral radiomic model built with the MLP classifier; (E) 10-mm peritumoral radiomic model built with the MLP classifier; (F) 10-mm peritumoral radiomic model built with the Naive Bayes classifier; (G) combined intratumoral and peritumoral radiomic model (Rad_MLP) built with the MLP classifier; (H) combined image feature model built with the SVM classifier; (I) multizone radiomic model built with the MLP classifier.

For each 2×2 confusion matrix, rows denote the ground truth of DEL status (0: DEL-negative; 1: DEL-positive), and columns denote the model-predicted DEL status (0: predicted DEL-negative; 1: predicted DEL-positive). The color intensity of each cell is proportional to the absolute number of samples, as indicated by the vertical color bar on the right.

Supplementary Figure 3. Confusion matrices of six predictive models for DEL status classification in the validation cohort

Panels A to F correspond to models constructed with distinct feature sets and classifiers: (A) intratumoral radiomic model built with the LightGBM classifier; (B) deep learning (DL) feature-based model built with the KNN classifier; (C) clinical feature-based model built with the logistic regression (LR) classifier; (D) combined radiomic model (Rad_MLP) built with the MLP classifier; (E) integrated model combining DL, clinical and radiomic features built with the KNN classifier; (F) stacking ensemble model built with the MLP classifier.

For each 2×2 confusion matrix, rows denote the ground truth of DEL status (0: DEL-negative; 1: DEL-positive), and columns denote the model-predicted DEL status (0: predicted DEL-negative; 1: predicted DEL-positive). The

color intensity of each cell is proportional to the absolute sample count, with the vertical color bar on the right indicating the numerical scale.

Supplementary Figure 4. Representative PET images of false-positive and false-negative cases from the Rad_MLP model in the validation cohort.

(A) A representative false-positive case (patient ID: PT54958). The patient was pathologically confirmed as non-DEL (ground truth = 0), while the Rad_MLP model yielded a DEL-predicted probability of 0.769 and misclassified the case as DEL-positive. (B) A representative false-negative case (patient ID: PT53701). The patient was pathologically confirmed as DEL-positive (ground truth = 1), while the Rad_MLP model yielded a DEL-predicted probability of 0.174 and misclassified the case as DEL-negative. For each case, coronal, sagittal, and axial PET views are presented from left to right, and the target tumor region of interest is highlighted in magenta.

Supplementary Table 5. Comprehensive diagnostic performance and calibration metrics of all candidate prediction models in the internal validation cohort

| Model | Accuracy | AUC（95% CI） | Sensitivity | Specificity | PPV | NPV | F1 | Threshold | Brier Score（95%CI） | PR AUC | Cohort |
| --- | --- | --- | --- | --- | --- | --- | --- | --- | --- | --- | --- |
| IT-only_LightGBM | 0.79 | 0.837（0.717 - 0.956） | 0.90 | 0.71 | 0.71 | 0.90 | 0.79 | 0.33 | 0.195（0.149 - 0.264） | 0.761 | Validation |
| Peri3mm_KNN | 0.74 | 0.804（0.676 - 0.932） | 0.58 | 0.88 | 0.79 | 0.72 | 0.67 | 0.40 | 0.202（0.126 - 0.295） | 0.759 | Validation |
| Peri5mm_LR | 0.72 | 0.726（0.570 - 0.882） | 0.68 | 0.75 | 0.68 | 0.75 | 0.68 | 0.28 | 0.234（0.155 - 0.319） | 0.700 | Validation |
| Peri5mm_MLP | 0.77 | 0.763（0.612 - 0.914） | 0.79 | 0.75 | 0.71 | 0.82 | 0.75 | 0.29 | 0.203（0.147 - 0.270） | 0.756 | Validation |
| Peri10mm_MLP | 0.81 | 0.836（0.709 - 0.962） | 0.84 | 0.79 | 0.76 | 0.86 | 0.80 | 0.35 | 0.179（0.133 - 0.231） | 0.812 | Validation |
| Peri10mm_NaiveBayes | 0.81 | 0.831（0.696 - 0.966） | 0.84 | 0.79 | 0.76 | 0.86 | 0.80 | 0.03 | 0.212（0.103 - 0.333） | 0.846 | Validation |
| Rad_MLP | 0.84 | 0.853（0.731 - 0.976） | 0.90 | 0.79 | 0.77 | 0.91 | 0.83 | 0.28 | 0.176（0.116 - 0.240） | 0.818 | Validation |
| combineimage_SVM | 0.74 | 0.632（0.435 - 0.828） | 0.42 | 1.00 | 1.00 | 0.69 | 0.59 | 0.55 | 0.201（0.141 - 0.271） | 0.728 | Validation |
| Multizone_MLP | 0.79 | 0.809（0.676 - 0.942） | 0.79 | 0.79 | 0.75 | 0.83 | 0.77 | 0.37 | 0.188（0.140 - 0.242） | 0.787 | Validation |
| Clinic_LR | 0.54 | 0.556（0.414 - 0.698） | 0.74 | 0.38 | 0.48 | 0.64 | 0.58 | 0.44 | 0.247（0.207 - 0.288） | 0.472 | Validation |
| DL+clinic_MLP | 0.84 | 0.868（0.759 -0.978） | 0.79 | 0.88 | 0.83 | 0.84 | 0.81 | 0.48 | 0.167（0.134 - 0.204） | 0.710 | Validation |
| Rad+clinic_MLP | 0.84 | 0.860（0.739 - 0.980） | 0.90 | 0.79 | 0.77 | 0.90 | 0.83 | 0.46 | 0.160（0.115 - 0.208） | 0.743 | Validation |
| DL_KNN | 0.70 | 0.774（0.640 - 0.908） | 0.68 | 0.71 | 0.65 | 0.74 | 0.67 | 0.40 | 0.199（0.129 - 0.274） | 0.680 | Validation |
| rad+DL+clinic_KNN | 0.74 | 0.800（0.670 - 0.931） | 0.53 | 0.92 | 0.83 | 0.71 | 0.65 | 0.60 | 0.181（0.116 - 0.259） | 0.769 | Validation |
| stacking_MLP | 0.86 | 0.818（0.658 - 0.978） | 0.74 | 0.96 | 0.93 | 0.82 | 0.82 | 0.50 | 0.154（0.102 - 0.218） | 0.876 | Validation |
| Ensemble | 0.44 | 0.143（0.013 - 0.272） | 1.00 | 0.00 | 0.44 | 0.00 | 0.61 | 0.21 | 0.176（0.130 - 0.229） | 0.889 | Validation |

Note. Values represent full performance metrics of all validation models in the internal validation cohort. 95% CIs for AUC were derived from the DeLong test; 95% CIs for PR-AUC and Brier score were estimated by bootstrap resampling with 2000 iterations. All thresholds were optimized by maximizing Youden’s index in the training cohort.

Abbreviations: AUC, area under the ROC curve; 95% CI, 95% confidence interval; PPV, positive predictive value; NPV, negative predictive value; PR-AUC, area under the precision-recall curve; IT, intratumoral; Peri, peritumoral; Rad, radiomics; DL, deep learning.

Supplementary Table 6. P values of the Hosmer–Lemeshow goodness-of-fit test for all prediction models in the training and internal validation cohorts

| Hosmer-Lemeshow | | | | | | |
| --- | --- | --- | --- | --- | --- | --- |
| set | Intratumoral_LightGBM | Peri3mm_KNN | Peri5mm_LR | Peri5mm_MLP | Peri10mm_MLP | Peri10mm_NaiveBayes |
| train | 0.42 | 1.00 | 0.92 | 0.668 | 0.22 | <0.001 |
| validation | 0.10 | 0.01 | 0.01 | 0.101 | 0.22 | <0.001 |
|  | combinefeatures_MLP | combineimage_SVM | Multizone_MLP | Clinic_LR | DL_KNN | DL+clinic_MLP |
| train | 0.45 | 0.623 | 0.17 | 1.00 | 0.91 | 0.002 |
| validation | 0.17 | 0.039 | 0.43 | 0.90 | 0.43 | 0.037 |
|  | combinefeatures+clinic_MLP | rad+DL+clinic_KNN | stacking_MLP | Ensemble |  |  |
| train | 0.05 | 0.94 | 0.09 | <0.001 |  |  |
| validation | 0.54 | 0.10 | 0.05 | <0.001 |  |  |

Note. Values in the table are P values of the Hosmer–Lemeshow test, with P > 0.05 indicating good model calibration. Abbreviations: Peri, peritumoral; LightGBM, light gradient boosting machine; KNN, k-nearest neighbor; LR, logistic regression; MLP, multilayer perceptron; DL, deep learning; rad, radiomics.

Supplementary Table 7. Ablation Study Results on the Independent Test Set (n=29)

(143 patients split 6:2:2 into training/validation/test; all models use XGBoost classifier)

| Model Configuration | Feature Composition | AUC (95% CI) | Accuracy | Sensitivity | Specificity | ΔAUC | P-value |
| --- | --- | --- | --- | --- | --- | --- | --- |
| A. Full Ensemble Model | Intra+Peri10mm + Clinic + DL | 0.866 (0.740-0.993) | 0.884 | 0.895 | 0.875 | — | — |
| B. Feature Type Ablation |  |  |  |  |  |  |  |
| B1. Remove Clinical | Intra+Peri10mm + DL | 0.816 (0.676-0.956) | 0.791 | 0.737 | 0.833 | -0.05 | 0.105 |
| B2. Remove DL | Intra+Peri10mm + Clinic | 0.772 (0.620-0.923) | 0.767 | 0.737 | 0.792 | -0.094 | 0.198 |
| B3. Radiomics Only | Intra+Peri10mm | 0.732 (0.577-0.889) | 0.721 | 0.737 | 0.708 | -0.134 | 0.096 |
| B4. DL Only | DL | 0.737 (0.583-0.890) | 0.721 | 0.737 | 0.708 | -0.129 | 0.08 |
| B5. Clinical Only | Clinic | 0.556 (0.414-0.698) | 0.535 | 0.737 | 0.375 | -0.31 | <0.001 |
| C. Spatial Region Ablation |  |  |  |  |  |  |  |
| C1. Intratumoral Only | Intra | 0.691 (0.526-0.855) | 0.674 | 0.579 | 0.75 | -0.175 | 0.021 |
| C2. Peritumoral Only (10mm) | Peri10mm | 0.765 (0.624-0.907) | 0.698 | 0.895 | 0.542 | -0.101 | 0.146 |
| C3. Intra + 3mm Peri | Intra+Peri3mm | 0.770 (0.625-0.914) | 0.767 | 0.632 | 0.875 | -0.096 | 0.162 |
| C4. Intra + 5mm Peri | Intra+Peri5mm | 0.715 (0.552-0.878) | 0.721 | 0.737 | 0.708 | -0.151 | 0.034 |
| D. Modality Ablation |  |  |  |  |  |  |  |
| D1. CT Only | Intra+Peri10mmCT | 0.754 (0.603-0.906) | 0.698 | 0.895 | 0.542 | -0.112 | 0.154 |
| D2. PET Only | Intra+Peri10mmPET | 0.816 (0.685-0.946) | 0.791 | 1 | 0.625 | -0.05 | 0.476 |

Notes:ΔAUC = AUC(model) − AUC(Full Ensemble Model A)；P-values calculated by DeLong test comparing each ablated model to Model A (bold: P < 0.05)；143 patients randomly split into training (n=86), validation (n=28), and test (n=29) sets (6:2:2 ratio)；All models used XGBoost classifier with identical random seed and data partitioning

Supplementary Table 8. NRI and IDI for incremental diagnostic value of the combined model relative to the clinical model

| Cohort | Model Comparison | NRI | IDI | IDI（P_value) |
| --- | --- | --- | --- | --- |
| Train | Combined vs Clinical | 0.677 | 0.328 | 0.019 |
| Validation | Combined vs Clinical | 0.634 | 0.262 | 0.211 |

Note. Values represent the incremental diagnostic performance of the combined model over the clinical-only model. Positive NRI and IDI values indicate improved performance; IDI P < 0.05 denotes statistical significance. Abbreviations: NRI, net reclassification improvement; IDI, integrated discrimination improvement.

Supplementary Table 9. Diagnostic performance and calibration comparison between the final radiomics model and conventional PET metabolic parameters in the training and internal validation cohorts

| Model | Accuracy | AUC（95% CI） | Sensitivity | Specificity | PPV | NPV | F1 | Threshold | hosmer_lemeshow | Cohort |
| --- | --- | --- | --- | --- | --- | --- | --- | --- | --- | --- |
| Rad_MLP | 0.80 | 0.914（0.863 - 0.966） | 0.97 | 0.70 | 0.65 | 0.98 | 0.78 | 0.27 | 0.453 | Train |
| SUVmax | 0.74 | 0.832（0.755 - 0.909） | 0.83 | 0.69 | 0.60 | 0.88 | 0.70 | 0.60 | 0.906 | Train |
| MTV | 0.75 | 0.774（0.684 - 0.864） | 0.56 | 0.86 | 0.69 | 0.78 | 0.62 | 0.55 | 7.39E-01 | Train |
| SUVmax+MTV+TLG | 0.78 | 0.827（0.747 - 0.908） | 0.64 | 0.86 | 0.72 | 0.81 | 0.68 | 0.58 | 0.339 | Train |
| Rad_MLP | 0.84 | 0.853（0.731 - 0.976） | 0.90 | 0.79 | 0.77 | 0.91 | 0.83 | 0.28 | 0.173 | Validation |
| SUVmax | 0.63 | 0.688（0.535 - 0.840） | 0.95 | 0.38 | 0.55 | 0.90 | 0.69 | 0.40 | 0.024 | Validation |
| MTV | 0.81 | 0.751（0.567 - 0.935） | 0.74 | 0.88 | 0.82 | 0.81 | 0.78 | 0.54 | 2.00E-04 | Validation |
| SUVmax+MTV+TLG | 0.70 | 0.658（0.484 - 0.832） | 0.37 | 0.96 | 0.88 | 0.66 | 0.52 | 0.60 | 0.202 | Validation |

Note. Values indicate diagnostic performance and calibration results of the final radiomics model and conventional PET metabolic parameters. All thresholds were optimized via the maximum Youden’s index in the training cohort. P values of the Hosmer–Lemeshow test > 0.05 represent good model calibration. Abbreviations: Rad, radiomics; MLP, multilayer perceptron; AUC, area under the ROC curve; 95% CI, 95% confidence interval; PPV, positive predictive value; NPV, negative predictive value; SUVmax, maximum standardized uptake value; MTV, metabolic tumor volume; TLG, total lesion glycolysis.

Supplementary Figure 5. Pairwise comparisons of diagnostic performance between the radiomics model and conventional PET metabolic parameters via the DeLong test

(A) Training cohort; (B) Internal validation cohort. The heatmap shows P values of pairwise DeLong tests comparing the AUC of the radiomics model with conventional PET metabolic parameters. A P value < 0.05 was considered to indicate a statistically significant difference in diagnostic performance. Abbreviations: Rad, radiomics; MLP, multilayer perceptron; SUVmax, maximum standardized uptake value; MTV, metabolic tumor volume; TLG, total lesion glycolysis; AUC, area under the ROC curve.

Supplementary Table 10. Subgroup performance of Rad_MLP in the internal validation set

| Subgroup | N | AUC (95% CI) | PR-AUC (95% CI) |
| --- | --- | --- | --- |
| Pathological subtype |  |  |  |
| GCB | 14 | 0.667 (0.284–1.000) | 0.647 (0.229–1.000) |
| Non-GCB | 29 | 0.924 (0.814–1.000) | 0.915 (0.773–1.000) |
| Disease site |  |  |  |
| Extranodal | 32 | 0.821 (0.641–0.965) | 0.772 (0.522–0.952) |
| Nodal | 11 | 0.821 (0.417–1.000) | 0.874 (0.618–1.000) |
| Ann Arbor stage |  |  |  |
| I–II | 19 | 0.843 (0.585–1.000) | 0.627 (0.316–1.000) |
| III–IV | 24 | 0.821 (0.601–0.985) | 0.855 (0.663–0.992) |

Note.CI, confidence interval; AUC, area under the receiver operating characteristic curve; PR-AUC, area under the precision-recall curve. All metrics were calculated on the internal validation set; 95% CIs were obtained using bootstrap resampling (2000 iterations).
